# Transparent, Ultra-Stretching, Tough, Adhesive Carboxyethyl Chitin/Polyacrylamide Hydrogel Toward High-Performance Soft Electronics

**DOI:** 10.1007/s40820-022-00980-9

**Published:** 2022-12-07

**Authors:** Jipeng Zhang, Yang Hu, Lina Zhang, Jinping Zhou, Ang Lu

**Affiliations:** 1 College of Chemistry and Molecular Sciences, Wuhan University, Wuhan, 430072 People’s Republic of China; 2 Hubei Engineering Center of Natural Polymer-Based Medical Materials, Wuhan University, Wuhan, 430072 People’s Republic of China

**Keywords:** Conductive hydrogels, Multifunctional sensors, Human–machine interaction, Electronic skin, Stretchable triboelectric nanogenerators

## Abstract

To date, hydrogels have gained increasing attentions as a flexible conductive material in fabricating soft electronics. However, it remains a big challenge to integrate multiple functions into one gel that can be used widely under various conditions. Herein, a kind of multifunctional hydrogel with a combination of desirable characteristics, including remarkable transparency, high conductivity, ultra-stretchability, toughness, good fatigue resistance, and strong adhesive ability is presented, which was facilely fabricated through multiple noncovalent crosslinking strategy. The resultant versatile sensors are able to detect both weak and large deformations, which owns a low detection limit of 0.1% strain, high stretchability up to 1586%, ultrahigh sensitivity with a gauge factor up to 18.54, as well as wide pressure sensing range (0–600 kPa). Meanwhile, the fabrication of conductive hydrogel-based sensors is demonstrated for various soft electronic devices, including a flexible human–machine interactive system, the soft tactile switch, an integrated electronic skin for unprecedented nonplanar visualized pressure sensing, and the stretchable triboelectric nanogenerators with excellent biomechanical energy harvesting ability. This work opens up a simple route for multifunctional hydrogel and promises the practical application of soft and self-powered wearable electronics in various complex scenes.

**Supplementary Information:**

The online version contains supplementary material available at 10.1007/s40820-022-00980-9.

## Highlights

- Hydrogel demonstrates superior merits of strain (1586%), self-adhesion (113 kPa for pigskin), high conductivity and transparency (92%).
- The wearable sensors with a gauge factor up to 18.54, wide pressure sensing range (0–600 kPa) enable the detecting, quantifying, and monitoring of human activities.
- The hydrogels were developed as electronic skin, high output stretchable CTA-TENGs and explored using as wearable keyboards for human-machine interaction.

**Supplementary Information:**

The online version contains supplementary material available at 10.1007/s40820-022-00980-9.

## Graphical abstract

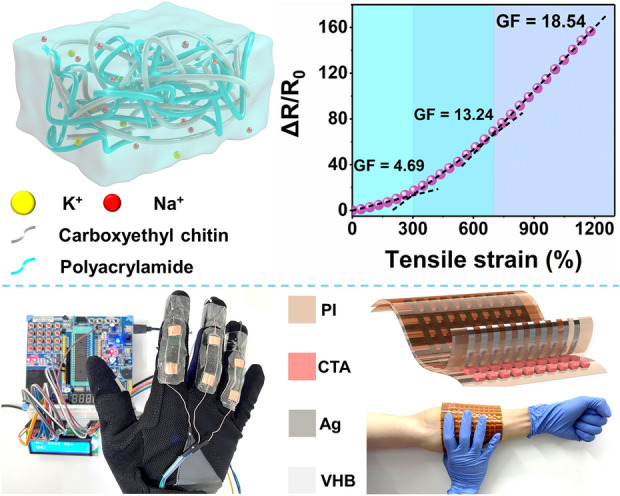

## Introduction

With the emergence of the era of big data, intelligent and soft electronics have attracted significant attention due to their potential application prospects in the Internet of Things (IoT), artificial intelligence (AI), electronic skins, and self-powered electronic devices [1–8]. The stretchability and mechanical compliance of these devices render their close assembly to the soft curvilinear contours of the human body, enabling the use of human-friendly soft electronics, such as tactile sensors, human–machine interactions, wearable power sources, and soft robotic/prosthetic/energy skins [9]. Correspondingly, the development of advanced sensors and generators to enable the imperceptible sensing and comfortable powering is highly desired for soft electronics.

Flexible strain sensors, which can convert mechanical stimuli into visible electrical signals in the form of signal transmission, exhibiting great potential in electronic skins, flexible touch screens, soft robotics, and so on [10, 11]. To facilitate interaction compliance and precise monitoring, next-generation strain sensors are expected to provide high stretchability, fast response, high sensitivity, and excellent repeatability in complex scenarios. Abundant efforts have been devoted on developing prototypical designs of diverse strain sensors with semiconductor materials [12], conductive nanomaterials [13, 14], hydrogels [15, 16], and liquid metals [17]. Among them, conductive hydrogels have aroused tremendous interests as emerging materials to prepare sensors for advanced sensing capabilities because of their specific advantages of the intrinsic stretchability, surface compliance, sustained conductivity, biocompatibility, and tailored mechanical property. For instance, a recent study reported a gelatin/NaCl organohydrogel-based strain sensor that enables sensitive electrical response under physical strain [18]. However, the low sensitivity (GF = 2.48) and unsatisfactory stretchability (maximum strain only up to 300%) of gel severely impeded its extensive application in strain sensors. Wang et al. [19] reported a class of self-adhesive strain sensors with conformal contact to skin for high-quality motion monitoring, indicating the crucial of good self-adhesive properties for strain sensors. On the other hand, it is difficult for some energy harvesters to achieve high stretchability, due to the intrinsic energy conversion mechanism, such as the strong magnetic field required for conventional electromagnetic generator, making them inconvenient for wearable electronics. Therefore, it is also crucial to design and develop ultra-stretchable, sustainable, and portable energy harvesters to efficiently convert ubiquitous ambient mechanical energy into electricity for practical applications in soft electronics.

Polysaccharide is a representative natural polymer with a rigid network due to the extensive hydrogen bonding and hydrophobic interaction among the molecular chains [20], which is regarded as one of the most promising raw materials to replace traditionally used nondegradable polymers in many occasions. Among these, chitin, the second most abundant renewable polymer after cellulose, exists mainly in shrimp and crab exoskeletons [21]. Here, we report the design and characterization of a class of noncovalently crosslinked multifunctional hydrogels with excellent comprehensive performance. Briefly, the hydrogels were prepared via facile heated polymerization of acrylamide monomer (AAm) in the presence of carboxyethyl chitin (CECT). The abundant noncovalent interactions including hydrogen bonds and hydrophobic interactions endow the hydrogels with excellent mechanical strength and toughness, as well as strong adhesion on various substrates. By controlling the ratio of the AAm and CECT, stretchability and mechanical toughness could be compromised, which makes it possible to prepare hydrogels with different properties for complex requirements. The hydrogel-based sensor shows high sensitivity and repeatable sensing signals for a wide range deformation in tension or compression and can be assembled into versatile electronics, including flexible tactile switch, electronic skin, and human–machine interactive system, to demonstrate the application potential for soft electronics. Furthermore, the hydrogel can also act as a stretchable electrode material (900% strain) to fabricate a sustainable triboelectric nanogenerator (TENG), which can achieve excellent electrical outputs. This work paves the way to the multifunctional hydrogel-based materials for the potential applications in high-performance soft electronics.

## Experimental Section

### Materials

Raw chitin was provided by Golden-Shell Biochemical Co., Ltd., Zhejiang, China, and the raw chitin powder was purified according to previous reported method [22]. Firstly, 100 g chitin powder was treated with 1 L 5 wt% NaOH solution for 10 h under vigorous stirring. This suspension was then filtered and washed with distilled water. Subsequently, the resulted chitin powder was treated with 1 L 7 wt% hydrochloric acid aqueous solution for 12 h to remove the residual protein. After filtration and rinsing with distilled water, the treated sample was dispersed in 1 L 5 wt% NaOH solution for 10 h. Finally, the treated chitin was treated with 4 wt% hydrogen peroxide for 4 h (pH 9, 80 °C), then washed with water and dried in vacuum oven to obtain purified chitin powder. Potassium hydroxide (KOH), urea, acrylamide (AAm, 98%), N, N’-methylene bis(acrylamide) (MBAA), and ammonium persulphate (APS) were obtained from Sinopharm Chemical Reagent Co., Ltd., Shanghai, China. The stretchable tape (VHB 4905) was purchased from 3 M Co., Ltd. (Shanghai, China). All reagents were used as received unless otherwise noted.

### Homogeneous Synthesis of Carboxyethyl Chitin (CECT)

Chitin is difficult to dissolve in commonly used solvents due to its highly ordered aggregate structure, extensive hydrogen bonding network, and negligible hydrophobic interactions between chitin chains, but it can be easily dissolved in KOH/urea aqueous solution via nonfreezing process according to our previous literature [5]. Firstly, chitin was dissolved in KOH/urea aqueous solution and then homogeneously modified with acrylamide by 1,4-conjugated addition reaction to obtain carbamoylethyl chitin (CMCT). Secondly, CMCT was hydrolyzed with NaOH solution and subsequently acetylated with hydrochloric acid to obtain CECT. The detail synthetic procedure is as follows. 8.0 g purified chitin powder was dispersed into 160 g 3.0 M KOH aqueous solution at room temperature for 48 h, and then 16 g urea and 224 g distilled water was added into the suspension, which was left for 48 h at − 20 °C. Next, the mixture was subjected to moderate stirring at room temperature, and dissolution of the chitin powder was achieved within 5 min. Further stirring for another 5 min led to a transparent and viscous chitin solution. After centrifugation at 10,000 rpm for 10 min, the chitin solution (400 g) was transferred into a 500-mL three-necked flask. Acrylamide solution (30.0 g in 20 mL of water) was added to the flask dropwise within 0.5 h at 15 °C. After reaction at 15 °C for another 12 h, the mixture was neutralized with concentrated HCl, dialyzed (Mw cutoff 3,500) against distilled water (3 d) and freeze-dried to obtain the carbamoylethyl chitin (CMCT). The CMCT was redissolved in NaOH aqueous solution (400 mL, 4.0 M) and stirred at room temperature for 12 h to hydrolyze the carbamoyl groups. The solution was neutralized with concentrated HCl, dialyzed (Mw cutoff 3500) against distilled water (3 d) and freeze-dried to obtain CECT.

The degree of substitution (DS) of carboxyethyl group (DS_COONa_, defined as the moles of carboxyethyl group per mole of glucose units) for CECT was determined by Eq. (1):

1 $$DS_{{{\text{COONa}}}} = \frac{{3A{\text{H8}} \times DA}}{{2A{\text{H9}}}}$$

where *A*_H8_ represents the integral area of two middle methylene protons (H8) from carboxyethyl group and *A*_H9_ represents the integral area of methyl protons (H9) from acetamido.

### Preparation of CECT/PAM Hydrogels (CTA)

CTA hydrogels were synthesized via a facile one-step polymerization. The composition of gels was referred to as C_x_T_y_A_z_, where C, T, and A represent the molar ratio of feed during the synthesis of carboxyethyl chitin (CECT), CECT, and acrylamide (AAm), respectively, x is the molar ratio of AAm to glucose units, y is the CECT weight ratio (wt%) in the pre-polymerization solution, and z is the AAm weight ratio (wt%) in the pre-polymerization solution. For example, C_11_T_4_A_20_ meant the molar ratio of AAm to glucose units was 11, the concentration of CECT was 4.0 wt%, and the concentration of AAm was 20 wt% in feed. Briefly, for synthesis C_11_T_4_A_20_, CECT-11 (0.6 g), AAm (3.0 g), MBA (0.0015 g, 0.05 wt% of AAm), APS (0.06 g, 2.0 wt% of AAm), and 11.4 g distilled water were added into a tube, and after stirring for 1 h, all the mixture was dissolved. Then a transparent solution was obtained. Finally, the resulting solution was poured into glass modules at 60 °C for 3 h to form gel. The pristine PAAm gel (C_0_T_0_A_20_) was prepared using the same gel preparation method, except no CECT was added. The use of different molds enables to produce different shapes of gels.

### Fabrication and Characterization of the Hydrogel-Based Single-Electrode TENG

The obtained hydrogel and two VHB films were used as electrode and triboelectrically charged layers, respectively. A hydrogel-based single-electrode mode TENG (CTA-TENG) was assembled by sandwiching the hydrogel electrode between the two VHB films. A piece of Ag foil was attached to the hydrogel electrode as the electric connector for measuring the electrical performances. The output performance of the CTA-TENG was characterized by applying a vertical force. The CTA-TENG was periodically pressed using a linear motor (P01_37X120_C_C1100, LinMot). The open-circuit voltage (*V*_OC_), short-circuit quantity (*Q*_SC_) and short-circuit current (*I*_SC_) during the contact-separation motion were measured using Keithley electrometer 6514. The output power density (*P*_out_) of the CTA-TENG was characterized by Eq. (2):

2 $$P_{out} = \frac{{I_{out}^{2} R_{load} }}{A}$$

where *I*_out_, *R*_load_, and *A* are the output current, the loaded resistance, and the contact area of the CTA-TENG, respectively.

### Equilibrium Swelling Ratios of Hydrogel Samples

The as-obtained hydrogel samples were swollen in distilled water until reaching an equilibrium at room temperature. Subsequently, the swelled samples were taken out from the distilled water and immediately weighed after sucking off the surface water with filter paper. The equilibrium swelling ratio was calculated according to Eq. (3):

3 $${\text{Equilibrium swelling ratio }}\left( \% \right) \, = \frac{{W_{{\text{W}}} - W_{{0}} }}{{W_{{0}} }} \times 100$$

where *W*_0_ and *W*_W_ represent the weight of hydrogels before and after swelling in water, respectively.

## Results and Discussion

### Design Principle and Synthesis of Carboxyethyl Chitin/Polyacrylamide Hydrogels

**Fig. 1:**
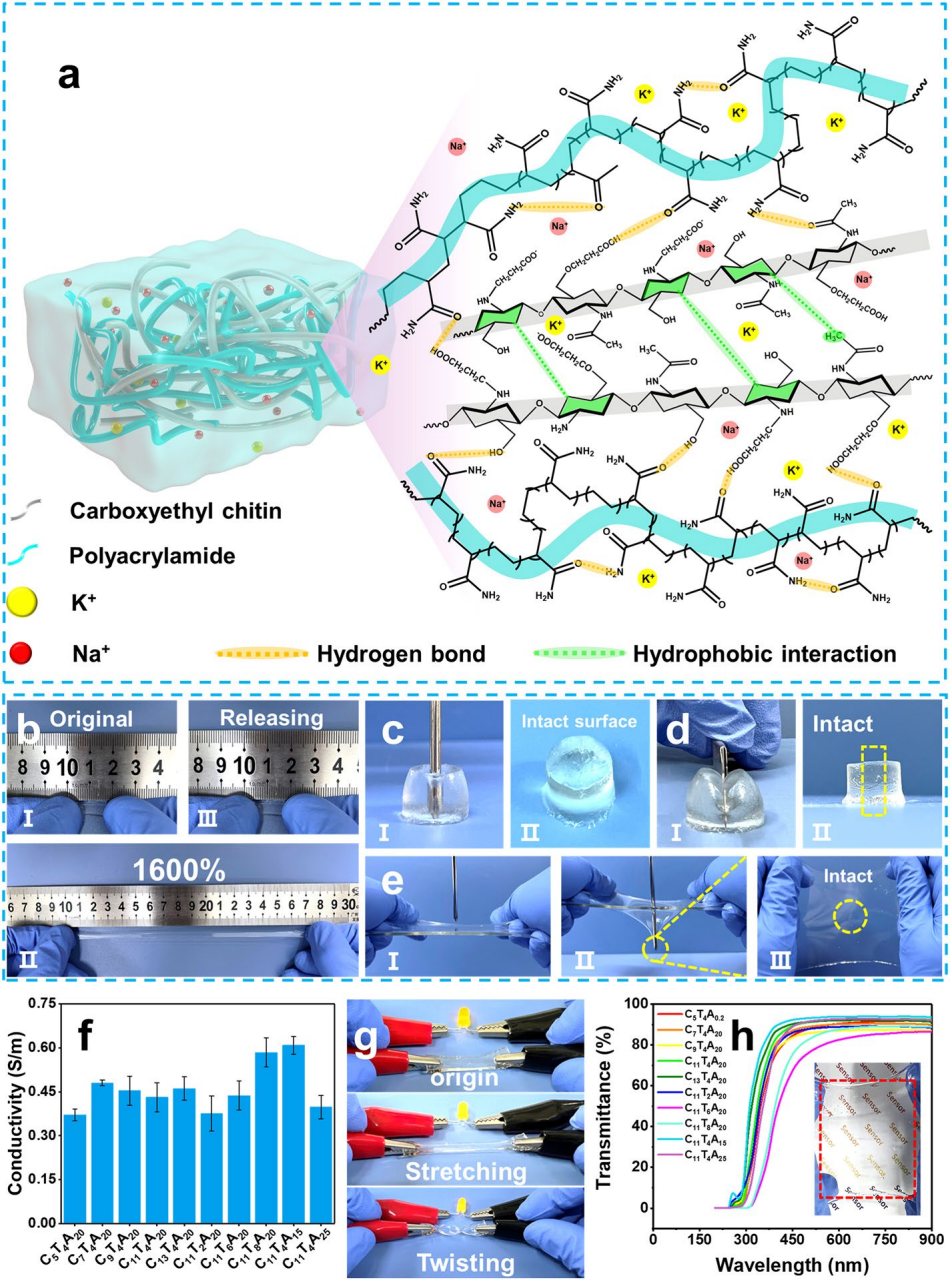
**a** Schematic illustration and molecular interaction among CECT, PAAm in the CTA hydrogel. **b** Photographs of the CTA hydrogel presenting neglectable residual strain without breaking even after being stretched to 1600% strain. **c** Photographs of poking a cylindrical CTA hydrogel with a sharp cross screwdriver. **d** Photographs of cutting a 1.8-cm-thick CTA hydrogel from top to bottom with a sharp blade. **e** Photographs of poking a stretched CTA hydrogel film with a sharp cross screwdriver. **f** Conductivity of the CTA hydrogels with different contents. **g** Luminance variations of LEDs using C_11_T_4_A_20_ hydrogel as a conductor which is subjected to stretching of 200% strain and twisting. **h** Transmittance of the CTA hydrogel with a thickness of 2 mm in the visible wavelength range of 400−900 nm. Inset is a photograph of a transparent C_11_T_4_A_20_ hydrogel

The design principle of carboxyethyl chitin/polyacrylamide (CTA) hydrogels with excellent mechanical properties, adhesion, high conductivity, long-term stable, and transparence by multiple noncovalent bond interactions is intuitively depicted in Fig. 1a. Carboxyethyl chitin (CECT) was selected due to the structural units of N-acetyl-D-glucosamine and D-glucosamine units, which possess abundant hydrophilic groups (hydroxyl, carboxy and amino groups) as well as hydrophobic pyranose ring and acetyl groups. These functional units can potentially interact with polyacrylamide (PAAm) through noncovalent bonding, including hydrogen bonding and hydrophobic interactions, etc., leading to enhanced mechanical properties. In addition, the CECT is well known for its excellent biocompatibility [23], which favors bioelectronic applications. The detailed preparation process can be found in Experimental Section. Briefly, chitin was dissolved in KOH/urea aqueous solution at low temperature and homogeneously modified with acrylamide by 1,4-conjugated addition reaction to obtain carbamoylethyl chitin (CMCT). Secondly, CMCT was hydrolyzed into CECT in alkaline medium, as depicted in Scheme S1. The ^1^H NMR spectrum of CECT in D_2_O (Fig. S1) showed that the signals at 2.47 ppm from the protons of carboxyethyl group appeared in CECT, validating that carboxyethyl was successfully grafted onto the CECT chains. Notably, the degree of substitution of carboxyethyl group of CECT (DS_COONa_) increased with an increase in the feed ratio of AAm to glucose units (Table S1), and all CECT has good water solubility. Then, AAm, N, N’-methylene bis(acrylamide) (MBAA) and CECT were dispersed in water to form a homogeneous, stable, and transparent mixture. Subsequently, the thermal initiator of ammonium persulfate (APS) was added to initiate the polymerization of AAm and to form CTA hydrogel (Fig. S2). Specifically, PAAm as the main polymer network defines the elasticity and stretchability of the hydrogel, while the CECT mainly functions as the rigid skeleton, which provides numerous noncovalent crosslinking sites and greatly improves the mechanical properties.

To efficiently tune the mechanical properties of the CTA hydrogels, the molar ratio of AAm and glucose units and the mass content of CECT and AAm were systematically changed. The resultant CTA hydrogels were termed C_x_T_y_A_z_, where C stands for the molar ratio of feed during the synthesis of CECT, T for CECT, and A for AAm, respectively, while x represents the molar ratio of AAm to glucose units during the synthesis of CECT, y represents the concentration (wt%) of CECT in the pre-polymerization solution, and z represents the concentration (wt%) of AAm in the pre-polymerization solution (Table S2). Remarkably, the obtained CTA hydrogels showed superior strength, and toughness, compared with the pristine PAAm hydrogel. As shown in Fig. 1b, the CTA hydrogel exhibited an ultra-stretchability of 1,600% without breaking. Furthermore, the hydrogel with a thickness 1.5 mm and a width of 3.5 mm was capable of easily lifting a 200 g load without any damage (Fig. S3). Besides, the excellent robustness and toughness of the hydrogel was demonstrated by poking a cylindrical CTA hydrogel with a sharp cross screwdriver. As shown in Fig. 1c, the surface of the hydrogel remained intact after being poked, suggesting the high mechanical softness and energy dissipation of the CTA hydrogel to endure highly concentrated stress. We also used a sharp blade to forcibly cut a 1.8-cm-thick cylindrical CTA hydrogel from top to bottom (Fig. 1d). The hydrogel remained intact after cutting without obvious scar left or partial rupture. To further demonstrate its advantageous mechanical property, a stretched CTA hydrogel film was poked by a sharp cross screwdriver, and it recovered to its initial state after the extremely concentrated stress was released (Fig. 1e). Moreover, we compressed the cylindrical hydrogel to approximate one-fourth of its initial thickness. After unloading, the hydrogel fully recovered without any visible cracks (Fig. S4). These results highly demonstrate the outstanding mechanical performance of the CTA hydrogel.

During the preparation, potassium hydroxide (KOH) and sodium hydroxide (NaOH) were employed in the process of preparing CECT to introduce ionic conductive behavior. Energy-dispersive spectroscopy mapping images exhibit that Na and K elements are uniformly dispersed in the CTA system (Fig. S5). As depicted in Fig. S6, the ionic conductivity of the CTA hydrogel samples was determined by electrochemical impedance spectroscopy (EIS) method. The intercepts of EIS curves with the x-axis were regarded as the impedance of the samples. The conductivity of these CTA hydrogels ranged from 0.38 to 0.62 S m^−1^ (Fig. 1f), surpassing most previously reported conductive composites (Table S3). The electrical conducting characteristic of C_11_T_4_A_20_ hydrogel was further displayed by a circuit with a light-emitting diode (LED) under 9.0 V power supply (Fig. 1g). In the stretching/twisting processes, no obvious change in brightness and the corresponding current values were observed (Fig. S7), even under a 200% strain or a large flexion angle of 720°, revealing the conductivity stability. Meanwhile, the as-prepared CTA hydrogels with a thickness 1.7 mm also exhibited high transparency (Fig. 1h), with the transmittance of the C_11_T_4_A_20_ hydrogel reaching 92.3% at the wavelength of 550 nm.

### Interactions in the CTA Hydrogels

**Fig. 2:**
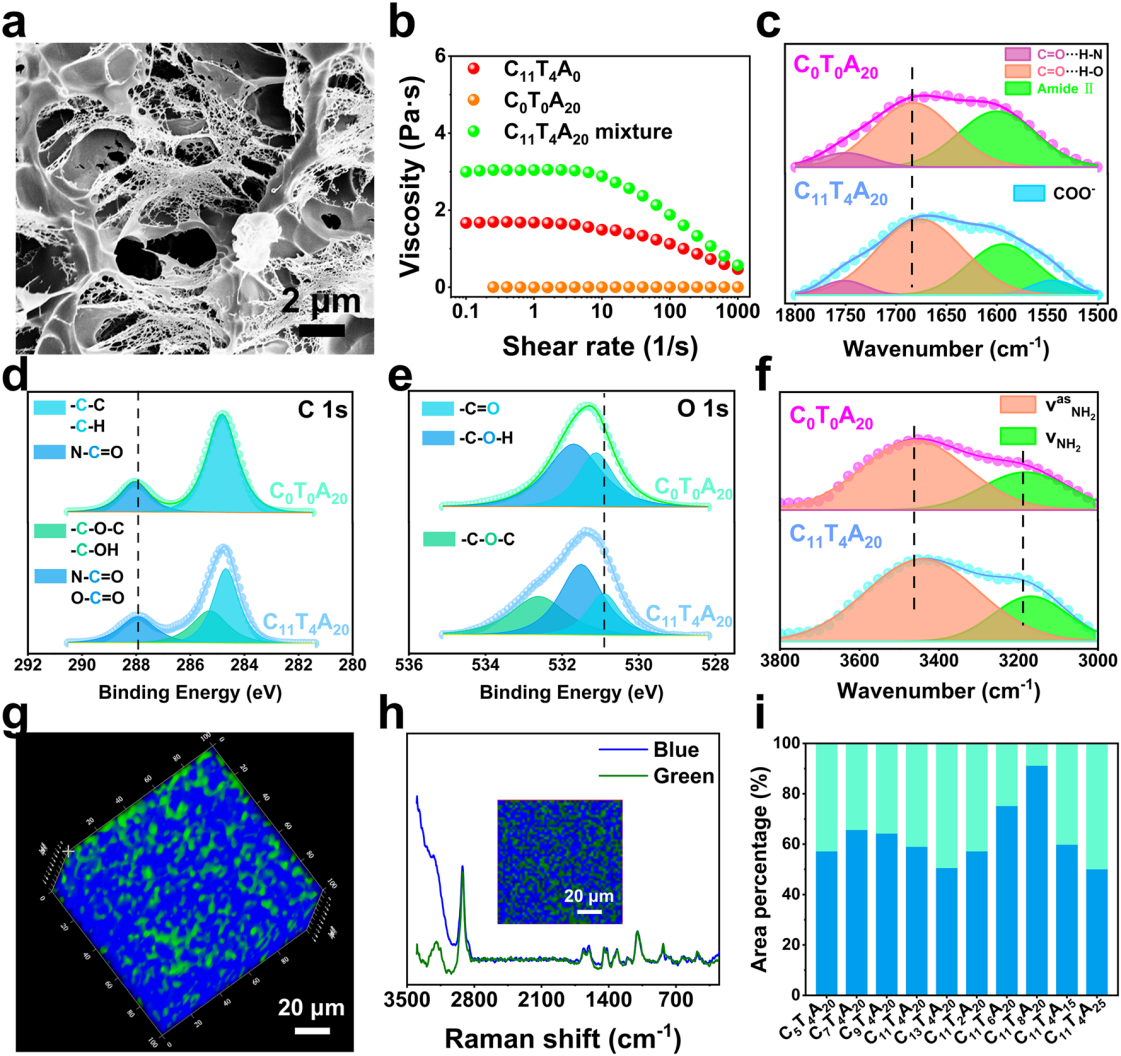
**a** SEM images of the C_11_T_4_A_20_ hydrogel. **b** The viscosity versus shear rate of C_11_T_4_A_0_, C_0_T_0_A_20_ and C_11_T_4_A_20_ mixture. **c** FT-IR spectra (1500–1800 cm^−1^) of C_0_T_0_A_20_ and C_11_T_4_A_20_ hydrogels. **d** C 1*s* XPS spectra of C_0_T_0_A_20_ and C_11_T_4_A_20_ hydrogels. **e** O 1*s* XPS spectra of C_0_T_0_A_20_ and C_11_T_4_A_20_ hydrogels. **f** FT-IR spectra (3000–3800 cm^−1^) of C_0_T_0_A_20_ and C_11_T_4_A_20_ hydrogels. **g** 3D Raman images of C_11_T_4_A_20_ hydrogel. **h** Raman spectra of the hydrophilic (blue) and hydrophobic (green) domains within the C_11_T_4_A_20_ hydrogel. The inset is the reconstructed Raman image of the hydrophilic and hydrophobic domains within the hydrogels obtained from the –OH and –NH stretching mode intensities (3000–3400 cm^−1^). **i** The percentage of hydrophilic (blue) and hydrophobic (green) domains of the hydrogels with different weight ratios of CECT to AAm

Figures 2A and S8 show the scanning electron microscopy (SEM) images of the CTA hydrogels with the interconnected 3D polyporous microstructure. Compared with the C_0_T_0_A_20_ hydrogel containing solely PAAm (Fig. S9), the participation of CECT had an effective influence on narrowing the pore distribution and increasing crosslinking density. Obviously, CECT supplied physical crosslinking junctions in the CTA system, further confirming the formation of a vigoroso crosslinked network is reliable for enhancing the mechanical damage resistance.

The interactions between CECT and PAAm were initially reflected by the zero-shear viscosity of the CECT and AAm mixture. Noticeably, the C_11_T_4_A_20_ mixture (3.04 Pa s) solution exhibited a higher viscosity than the C_11_T_4_A_0_ (1.65 Pa s) (Fig. 2b), which suggested the existence of CECT-CECT and CECT-AAm noncovalent interactions within the C_11_T_4_A_20_ mixture. Fourier transform infrared (FT-IR) spectroscopy and X-ray photoelectron spectroscopy (XPS) of C_11_T_4_A_20_ and C_0_T_0_A_20_ hydrogels were then performed to identify the noncovalent interactions within the CTA hydrogel, as illustrated in Fig. 2c. The C = C stretching vibration at 1612 cm^−1^ from AAm disappeared (Fig. S10), indicating that the polymerization occurred [24]. The significant characteristic peaks at 1751, 1684, and 1601 cm^−1^ are attributed to the –C=O···H–N–, –C=O···H–O– stretching vibration (amide I), and –N–H bending vibration (amide II) of the amide group, respectively [25]. The red-shift of the absorption band at 1675 cm^−1^ in C_11_T_4_A_20_ hydrogel compared with the C_0_T_0_A_20_ hydrogel verified the formation of –C=O···H–O– hydrogen bonding between CECT and PAAm. In addition, a new absorption band at 1543 cm^−1^ appearing in the C_11_T_4_A_20_ hydrogel was assigned for –COO^−^ stretching vibration compared with the C_0_T_0_A_20_, which was attributed to the strengthened interaction between CECT and PAAm. As shown in Fig. 2f, the C_0_T_0_A_20_ hydrogel showed two broad characteristic peaks at 3462 and 3185 cm^−1^ for –N–H stretching vibrations [25, 26]. After CECT introduction, these two characteristic peaks in C_11_T_4_A_20_ hydrogel were broadened and shifted to 3441 and 3169 cm^−1^, respectively, further suggesting the formation of hydrogen bonding between the CECT and PAAm [27]. The XPS spectrum of N 1* s* verified that C_11_T_4_A_20_ hydrogel was successfully synthesized (Fig. S11). Figure 2d, e gave the high-resolution C 1*s* and O 1*s* XPS spectra of C_0_T_0_A_20_ and C_11_T_4_A_20_. The new characteristic peaks of C–O–C/C–OH (285.28 eV) and C–O–C (532.64 eV) were observed in C_11_T_4_A_20_, which further evidenced the formation of C_11_T_4_A_20_ hydrogel. Notably, the C 1*s* spectra of C_0_T_0_A_20_ can be deconvoluted into two characteristic peaks of C–C/C–H (284.81 eV) and N–C=O (288.04 eV), while the O 1*s* spectra included two characteristic peaks of C=O (531.12 eV) and C–O–H (531.70 eV). Moreover, after the addition of CECT, the peaks of N–C=O and C=O in C_11_T_4_A_20_ shifted to lower binding energy (from 288.04 to 287.85 eV and from 531.12 to 530.89 eV, respectively) compared with C_0_T_0_A_20_, which confirmed the strong hydrogen bonding interaction between the hydroxyl group, amino group, and carboxyl group of CECT and the amino group of PAAm [28].

Swelling tests were conducted to calculate the equilibrium swelling ratio for evaluating the density of the hydrogen bonding network among the CTA hydrogels. As illustrated in Figs. S12 and S13, the C_11_T_8_A_20_ hydrogel displayed the highest equilibrium swelling ratios among the CTA samples, which might result from the increasing mass fraction of CECT caused the hydrogel to contain more hydrophilic groups, and more hydrogen bonding network. To investigate the multiple noncovalent crosslinking mechanism within the CTA hydrogel, we further used 3D Raman spectroscopic imaging to determine the distribution of the structural domains [28–31]. The spatial resolution of the Raman mapping was limited to 100 nm, thus preventing the identification of the exact positions of the different interaction domains in the CTA hydrogel. A uniform distribution of two different local chemical environments (blue and green colors) can be observed in the C_11_T_4_A_20_ hydrogel (Fig. 2g), clearly demonstrating macroscopically homogeneous but microscopically separated domains within the C_11_T_4_A_20_ hydrogels. In addition, multivariate curve resolution (MCR) was applied to evaluate the proportions of the different noncovalent interactions within the CTA hydrogel (Fig. 2h and S14). The reconstructed Raman images revealed the respective intensities of the vibrational stretching for -OH and -NH (3000–3400 cm^−1^) in blue and green colors. The micro-scale pronounced intensities of the blue spectrum came from the hydrophilic (hydrogen bonding) crosslinked domains (-OH and -NH- rich regions), whereas the green one corresponded to the relatively hydrophobic crosslinked domains (-OH and -NH- poor regions). The proportion of the hydrophilic crosslinked domains are shown in Fig. 2i, which is roughly consistent with the result of swelling tests.

### Mechanical Properties of the CTA Hydrogels

Mechanical properties of CTA hydrogels were quantitatively analyzed to explore the effect of the multiple noncovalent interactions. By tuning the molar ratio of AAm and glucose units (from 5:1 to 13:1), the mass fraction of the CECT (from 2 to 8 wt%) and AAm (from 15 to 25 wt%), a series of CTA hydrogels with different mechanical properties were obtained. As shown in Table S2, all the tested hydrogels exhibited good stretchability (tensile strain of 835.57−1585.77%), mechanical strength (maximum tensile stress of 121.26−226.84 kPa, maximum compressive stress of 0.38−3.32 MPa) and fatigue resistance.

**Fig. 3:**
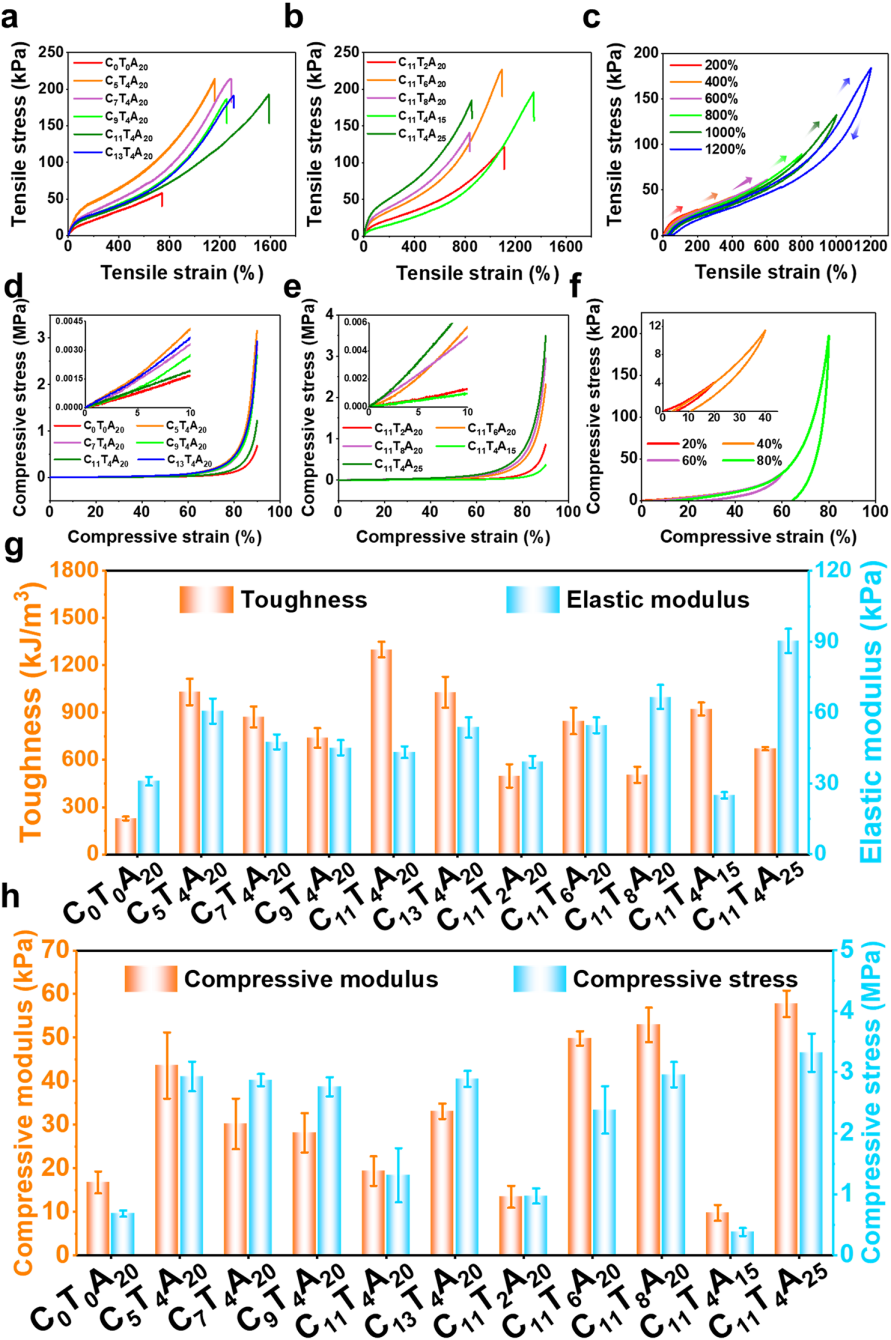
Tensile stress–strain curves of CTA hydrogels with different: **a** molar ratio of AAm to glucose units and **b** mass fraction of CECT and AAm. **c** Representative cyclic tensile stress–strain curves of C_11_T_4_A_20_ in various strains ranging from 200 to 1200%. Compressive stress–strain curves of CTA hydrogels with different: **d** molar ratio of AAm to glucose units and **e** mass fraction of CECT and AAm. The insets present the results over an initial narrow strain range. **f** Representative cyclic compressive stress–strain curves of C_11_T_4_A_15_ in various strains ranging from 20 to 80%. **g** Tension and **h** compression properties of CTA hydrogels

With the mole ratio of AAm and glucose units increased from 5:1 to 11:1, the tensile strain of CTA hydrogels was raised from 1157.73 to 1585.77%, the tensile stress changed from 186.41 to 213.93 kPa, and the toughness changed from 739.70 to 1299.71 kJ m^−3^, respectively (Fig. 3a, g). The tensile strain, tensile stress, and toughness of the C_11_T_4_A_20_ hydrogel were approximately 2, 4, and 6 times higher than that of the C_0_T_0_A_20_ hydrogel. The enhancement of mechanical properties is attributed to the formation of a densely noncovalent network. Then as the mole ratio of AAm and glucose units was further increased to 13:1, the tensile strain and toughness significantly decreased to 1307.11% and 1030.17 kJ m^−3^, respectively. As we fixed the mole ratio of AAm and glucose units and increased the mass fraction of the CECT from 2 to 8 wt%, the tensile strain of the CTA hydrogels decreased from 1585.77 to 835.57%, whereas the elastic modulus were significantly enhanced from 39.16 to 66.62 kPa (Fig. 3b, g). A similar trend can be observed with the mass fraction of the AAm increased from 15 to 25 wt%. The results might be attributed to the fact that the increased CECT and AAm led to enhanced noncovalent crosslinking interaction and dense hydrogel network [32–34]. Among the CTA hydrogels, C_11_T_4_A_20_ showed the highest toughness of 1299.71 kJ m^−3^ with the largest tensile strain of 1585.77% and comparable modulus to the human tissue (around 1−100 kPa) [35], and thus the C_11_T_4_A_20_ hydrogel could achieve a more comfortable human–machine interface interaction. The viscoelasticity of the C_11_T_4_A_20_ hydrogel was further investigated by cyclic tensile tests, from which the residual strain and the energy dissipation could be obtained. As depicted in Fig. 3c, the closed stress–strain curves of the C_11_T_4_A_20_ with pronounced hysteresis loops and residual strains at different strains were recorded. The appearance of hysteresis loops in the loading–unloading cycle of the tensile tests was related to the partial breaking of the abundant noncovalent interactions in the hydrogels, including the hydrogen bonding and the hydrophobic interactions in this case. This phenomenon of energy dissipation is commonly observed in gels with noncovalent interactions [24, 36]. When the strain was increased from 200 to 1200%, the dissipated energy increased from 5.26 to 126.78 kJ m^−3^ (Fig. S15a), suggesting the effective energy dissipation at large strains. We also observed the residual strain is about 50% even at a large tensile strain (Fig. S15b). It was also noteworthy that the hydrogels exhibited good fatigue resistance in the consecutive tensile test. Figure S16a shows the 15th cycle loading − unloading curves of the C_11_T_4_A_20_ without a resting interval at a maximum tensile strain of 400%, and the curves recorded in different cycles almost overlapped with each other after the first cycle [37], which was a vital feature for the strain-sensing function [38]. The corresponding dissipation energy decreased between the first and second laps but nearly remained constant from the third lap (Fig. S16b), which illustrate that the damaged network could be reconstructed during the tensile cycles [39, 40]. The first loading/unloading curve and curves after being stretched to 400% with various recovery times between two cycles were compared to characterize the self-recovery property. As shown in Fig. S17, with the extension of the time interval, the dissipated energy and maximum stress of the hydrogel gradually recovered. After 15 min, the dissipated energy and maximum stress of the stretched C_11_T_4_A_20_ hydrogel recovered to 15.77 kJ m^−3^ and 42.44 kPa, approaching its original values (17.49 kJ m^−3^ and 44.65 kPa). The excellent self-recovery ability of the C_11_T_4_A_20_ hydrogel might have benefited from the reversible noncovalent interaction between CECT and PAAm chains, whose mechanism was similar to that of the ionic hydrogels due to the existence of the reversible interaction within the network [41].

Compressing measurements (Fig. 3d, e and h) were performed and exhibited similar enhancement behaviors, whereby the CTA hydrogels presented remarkable toughness with a 0.6–4.8 times higher compressive stress, 0.6–3.5 times higher compressive modulus and 0.5–5.5 times higher fracture energy than the C_0_T_0_A_20_ hydrogel, indicating that the abundant noncovalent bonds significantly improved the mechanical properties of hydrogels. The higher modulus normally led to more brittleness and thus worse resilience [42]. The lowest compressive modulus occurred in the C_11_T_4_A_15_ sample (9.76 kPa), wherein the compressive stress and fracture energy were 0.38 MPa and 24.65 kJ m^−3^, respectively. As demonstrated in Fig. 3f, the C_11_T_4_A_15_ sample exhibited obvious hysteresis loops during the loading−unloading cycle, and the hysteresis loop enlarged with the increase in pressure. It was found that the dissipated energy increased from 0.12 kJ m^−3^ at a strain of 20% to 24.61 kJ m^−3^ at a strain of 80% (Fig. S18), which was due to the destruction of noncovalent interactions in the hydrogel network caused by deformation. Next, the continuous 15 loading–unloading cycle tests of the C_11_T_4_A_15_ sample upon a 70% compressive strain were carried out (Fig. S19a). No obvious shifting and breaking were observed in the whole cyclic process and the dissipated energy of C_11_T_4_A_15_ almost remained after the first cycle (Fig. S19b), indicating a good fatigue resistance and highly deformation tolerant performance [43, 44]. At 50% compressive strain (Fig. S20a), the unbroken noncovalent interaction drove the network back to the unpressed state and a 25-min rest interval resulted in 87% recovery (Fig. S20b). In summary, the CTA hydrogels with comprehensive mechanical properties manifested extraordinary stretchability and excellent toughness under different strains, showing a broad application prospect in flexible electronic devices.

### Self-Adhesive Properties of CTA Hydrogels

**Fig. 4:**
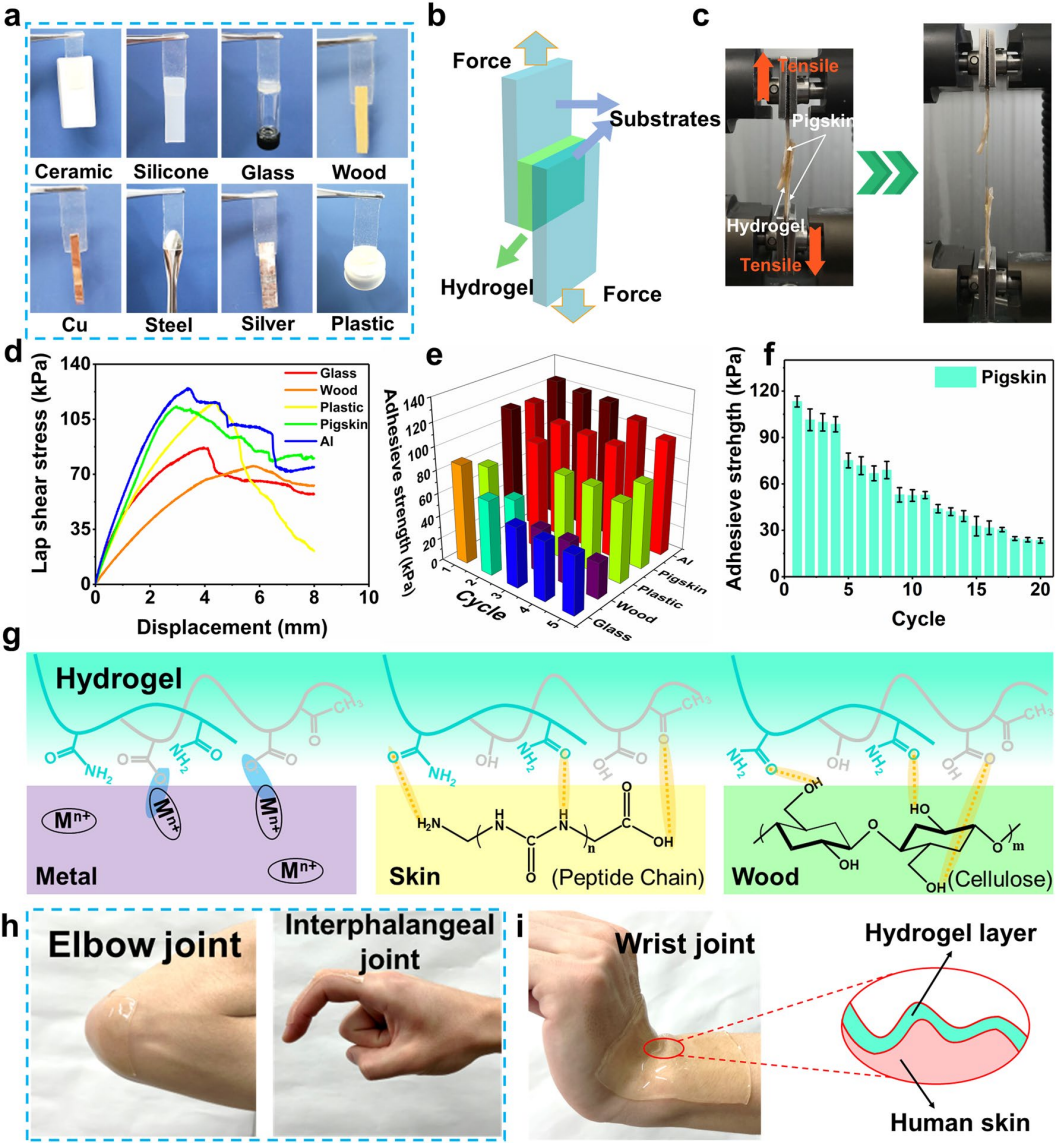
**a** Photographs of C_11_T_4_A_20_ hydrogel adhered on diverse substrates. **b** Schematic illustration of the lap shear test. **c** Photograph of lap shear test using pigskin as substrate. **d** Representative stress − displacement curves of lap shear tests on glass, wood, plastic, Al, and pigskin glued by C_11_T_4_A_20_ hydrogel. **e** Adhesive strength and reusability of C_11_T_4_A_20_ hydrogel on various substrates. **f** Adhesion durability and stability of C_11_T_4_A_20_ hydrogels on pigskin demonstrated by 20 successive peeling-off cycle tests. **g** Proposed adhesion mechanism between CTA hydrogel and various substrates. **h, i** Photographs of C_11_T_4_A_20_ hydrogels conformally self-adhered to the body’s frequently moving joints

The self-adhesiveness is the exceedingly favored property of the hydrogels, since the strong self-adhesive capability is vital for the interfacial connection with electrode layers, a crucial aspect for the functionality and reliability of flexible wearable hydrogel electronics [36, 45]. Given the abundant hydroxyl groups, amino groups, and carboxyl groups on CTA hydrogels, the C_11_T_4_A_20_ hydrogel possesses universal and self-adaptive adhesion on both hydrophilic and hydrophobic surfaces, including ceramic, silicone, glass, wood, Cu, steel, silver, and plastic, as demonstrated in Fig. 4a. To further verify the adhesive universality of C_11_T_4_A_20_ hydrogel, the adhesion strength was quantified by the lap shear test on assemblies with the hydrogel sandwiched between a pair of substrates (Fig. 4b). The maximum adhesive strength was taken as the interface adhesion strength for interface failure, representing the interface adhesion strength for interface invalidation, and the values of C_11_T_4_A_20_ hydrogel on glass, wood, plastic, pigskin, and Al were 86.9, 75.2, 115.9, 113.2, and 124.7 kPa at 3.9, 5.8, 4.4, 2.9, and 3.4 mm, respectively (Fig. 4d), which was higher than that most of the reported conductive hydrogels and gel electrolytes (Fig. S21).

According to the adhesion results, the hydrogels exhibited excellent endurance to shear, possibly attributed to the fact that the cohesive force of the hydrogels could dissipate energy during the peeling process [46]. Furthermore, repeatable adhesive ability on these substrates was achieved for our hydrogels, and 42−83% of the original adhesive strength remained after five detachment–reattachment cycles (Fig. 4e), attributed to the reversible hydrogen bonds and complexation interactions between the exposed hydroxyl groups, amino groups, and carboxyl groups and the substrate surface [47]. In addition, in order to evaluate the stability and durability of self-adhesiveness, 20 successive cycles of the lap shear testing for C_11_T_4_A_20_ hydrogel were carried out on pigskin (Fig. 4c), which exhibited constant adhesion to pigskin (25.4 kPa) even after 20 repeated peeling/adhering cycles, implying the outstanding adhesion repeatability (Fig. 4f). This decrease in adhesion strength may be due to the accumulated fracture on the hydrogel or contamination caused by dirt.

The high and durable self-adhesion of the C_11_T_4_A_20_ hydrogel is ascribed to the interaction of functional groups from the surface of hydrogels with substrates, where hydroxyl, amino, and carboxyl groups could form abundant interactions including strong hydrogen bonding and metal coordination as illustrated in Fig. 4g. Taking the wood as an example, the surface of the wood was mainly composed of hydroxyl groups and oxygen atoms, which could interact with hydroxyl, amino, carbonyl, and carboxyl groups by hydrogen bonding interaction. A similar mechanism also existed for the adhesion of C_11_T_4_A_20_ hydrogel to other substrates such as skin. As a visual observation, C_11_T_4_A_20_ hydrogel was able to achieve intimate contact with both skin during the vigorous swing of the hand (Video S1) and irregular curved skin surfaces such as elbow, wrist, and interphalangeal joints without any retraction or delamination during the testing, disclosing their strong interfacial adhesion (Fig. 4h, i). Notably, the conformal contact between hydrogel and skin even during body movement is demonstrated (Fig. 4i). The advantages become more remarkable for curvilinear skin and irregular change on the skin surface [19]. Overall, the excellent adhesive property of the CTA hydrogels guaranteed the effectiveness of the flexible electronic device assembly process and satisfied the demands of high-quality signals collection for wearable stretchable strain sensors.

### Sensing Properties of CTA Hydrogels

**Fig. 5:**
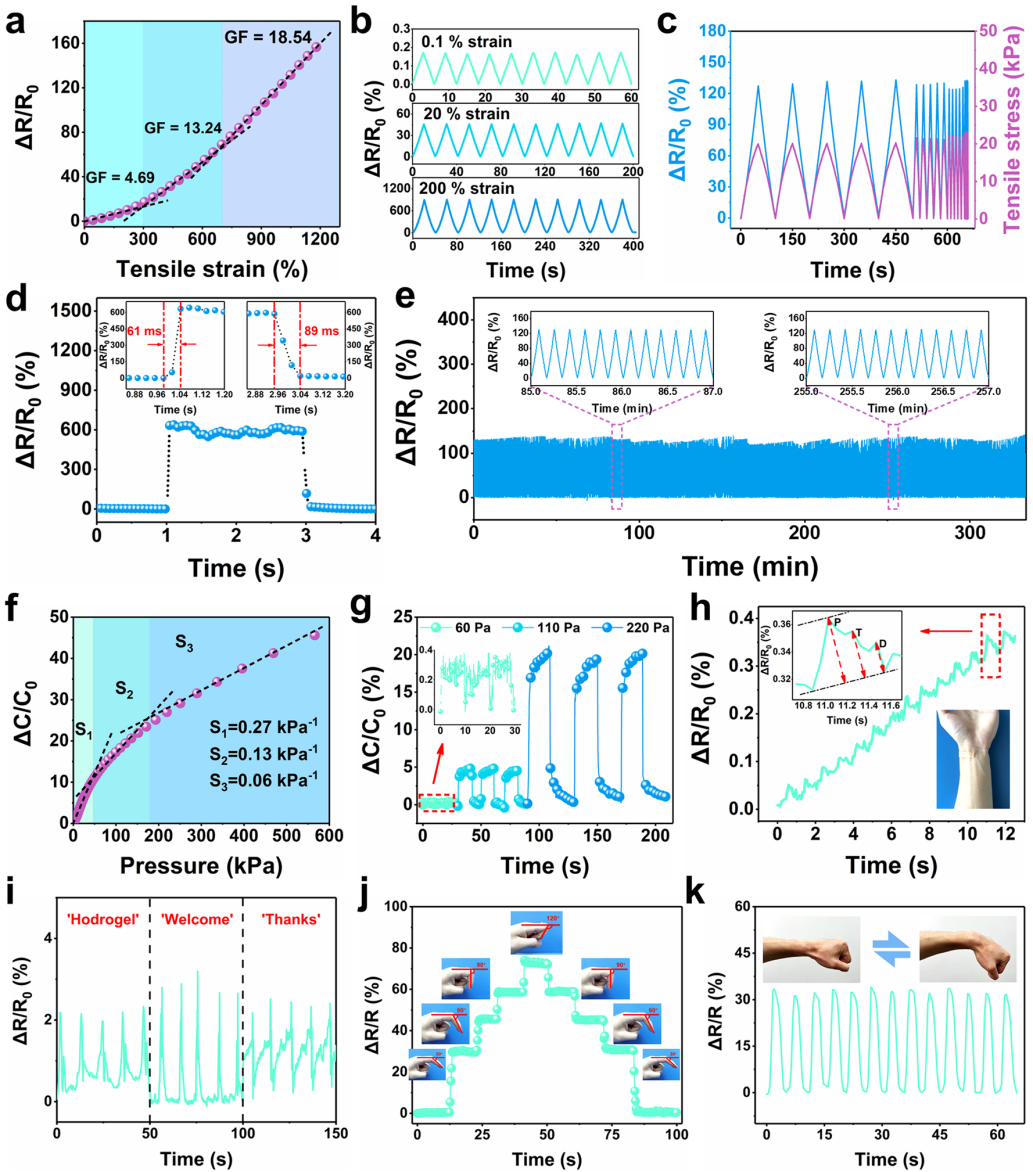
**a** Relative resistance changes of C_11_T_4_A_20_ sensor as a function of tensile strain. **b** Relative resistance changes of C_11_T_4_A_20_ sensor under different tensile strains during 10 successive cycles. **c** Relative resistance changes under tensile stress with various frequencies. **d** Response time and release time of C_11_T_4_A_20_ sensor. **e** Relative resistance changes under repeated loading–unloading processes with a strain of 50% for 2000 cycles, showing the durability of the sensor. **f** Relative capacitance changes of C_11_T_4_A_15_ sensor versus the applied pressure. **g** Relative capacitance changes of C_11_T_4_A_15_ sensor under different compressive stress. **h** Relative resistance changes of C_11_T_4_A_20_ strain sensor monitoring wrist pulse. Inset shows the photograph of a sensor attached to the wrist. **i** Phonation when the wearer spoke “Hydrogel,” “Welcome,” and “Thanks.” **j** Relative resistance changes with bending of finger. **k** Repeated bending/unbending movements of signal monitoring of the wrist

The unique combination of superior mechanical property, high ionic conductivity, and excellent adhesive property endows the potential application of the CTA hydrogel as high-performance wearable strain sensors. The relative resistance variation (Δ*R*/*R*_0_, where Δ*R* is the relative change in resistance, *R*_0_ is the original resistance when unstretched) of the C_11_T_4_A_20_ hydrogel-based sensor with the tensile strain is depicted in Fig. 5a, in which the Δ*R*/*R*_0_ value increased monotonously with the increase in tensile strain in the whole stretching process. This strain-resistance effect was attributed to the variation in the 3D network structure for electron transport and the change of porous microstructure for ions conduction during deformation. In the initial state, free ions are uniformly distributed in the hydrogel, forming a complete 3D conductive pathway for electron transportation. And the porous microstructure in the C_11_T_4_A_20_ hydrogel facilitated the transmission of free ions. Subsequently, the inhomogeneity of the hydrogel matrix gradually increased during low stretching and the decrease in the cross-sectional area of the C_11_T_4_A_20_ hydrogel further slowed down the free ion conduction, which led to the gradually increased resistance. When hydrogel suffered from large tensile deformation, the ion concentration per unit volume changed greatly, which might damage the electron transport between ions. And the cross-sectional area of the C_11_T_4_A_20_ hydrogel was further reduced, making the free ion conduction slower. Thus Δ*R*/*R*_0_ value increased sharply. The sensitivity of the C_11_T_4_A_20_ hydrogel-based sensor could be evaluated by using GF, defined as GF= (Δ*R*/*R*_0_)/ε (ε is the strain). As shown in Fig. 5a, the relative resistance change of the C_11_T_4_A_20_ was split into three linear response sections, including 0–300% with a GF of 4.69, 300–700% with a GF of 13.24, and 700–1248% with a GF of 18.54, respectively, indicating that the C_11_T_4_A_20_ hydrogel-based strain sensors with high sensitivity can be used in a very wide working strain range.

It can be concluded that the C_11_T_4_A_20_ sensor outperforms the traditional brittle semiconductors or metal foils (GF ≈ 2, ε < 5%) in light of both sensing strain range and GF [12]. The outstanding sensitivity at both small and large strains, along with the exceedingly broad sensing range, enables the C_11_T_4_A_20_ sensor to monitor and identify the full-range human activities. Table 1 shows the detailed comparison of GF and working strain range between C_11_T_4_A_20_ sensor and other gel sensors reported in previous studies. Furthermore, the sensing performances of the C_11_T_4_A_20_ sensor under different strains during the stepwise cyclic tensile deformation were investigated (Figs. 5b and S22). Profiting from the good sensitivity in a small strain range, the strain detection limit can be as minute as 0.1%, and the output signal was highly reproducible at a minute 0.1% strain with ∆*R*/*R*_0_ = 0.0016. As shown in Fig. 5c, the output resistance signal and the input stress of C_11_T_4_A_20_ matched well and both waveform peaks synchronized, indicating a negligible signal hysteresis. When a pressure of 21 kPa was applied with different frequencies (0.01–0.5 Hz), ∆*R*/*R*_0_ was kept consistent with its mechanical behavior and no electromechanical hysteresis was observed. A fast response to the applied strain was also observed, and the response time and recovery time were about 61 and 89 ms, respectively, as shown in Fig. 5d. The fast response was indisputably conducive to monitoring peoples’ fast and complex movements in real time, such as running and dancing. Moreover, the C_11_T_4_A_20_ sensor also presented rapid and excellent sensing response stability during 2000 cycles of 50% strain (Fig. 5e), and the dynamic cyclic response behavior of the encapsulated C_11_T_4_A_20_ sensor remained stable for 2 months (Fig. S23), indicating remarkable durability and impressive repeatability of the sensor during long-term loading–unloading processes.

**Table 1:** Comparison of the CTA-based sensor in this work with previously reported gel sensors

Materials	Gauge factor (GF)	Max. strain (%)	Response time	Application of human–machine interface	Reference
MWCNT/MoO_3_	46.3 (< 60%)	60	50 ms	No	[48]
PTCM-Gly organohydrogel	4.15 (< 250%) 8.21 (250–500%)	500	N/A	No	[49]
PVA-CNF organohydrogel	1.2 (< 150%) 1.5 (150–400%)	400	N/A	No	[50]
MASTA–PANI5	1.13 (< 110%) 1.61 (110–280%) 2.18 (280–400%)	400	N/A	No	[51]
HSAH/PHEAA SP-DN eutectogel	2.49 (< 100%) 6.29 (100–200%) 8.68 (200–300%) 10.47 (300–500%)	500	N/A	No	[52]
NaCl/SA/PAM hydrogel	2.0 (< 200%) 2.7 (200–1800%)	1800	N/A	No	[53]
SFRHs	0.82 (< 300%) 2.67 (300–1100%)	1100	N/A	No	[54]
TA@HAP NWs-PVA(W/EG)	2.84 (< 350%)	350	51 ms	No	[55]
HK-M-PAAm hydrogel	1.79 (< 100%) 5.72 (100–500%) 10.22 (500–1000%)	1000	130 ms	Yes	[56]
SGC	4.135 (< 200%) 8.015 (200–500%) 14.507 (500–1000%)	1000	163.1 ms	No	[57]
PAM/PBA-IL3/CNF2	3.41 (< 300%) 8.36 (300–1000%)	1000	195 ms	No	[58]
PAAN hydrgel	2.6 (< 300%) 7.8 (300–1210%) 17.9 (1210–1520%)	1520	N/A	No	[38]
Gelatin/NaCl organohydrogel	0.75 (< 50%) 2.48 (50–200%)	200	N/A	Yes	[18]
XSBR/SSCNT	4.24 (< 170%) 25.98 (170–214%)	214	200 ms	Yes	[7]
LM/TPU	1.35 (< 100%) 2.69 (100–200%)	200	N/A	Yes	[59]
***CTA hydrogel***	***4.69 (< 300%)*** ***13.24 (300–700%)*** ***18.54 (700–1248%)***	***1248***	***61 ms***	***Yes***	***This work***

The significance of bolditalic is to stand out conspicuously

Besides the responses to tensile strains, the C_11_T_4_A_15_ hydrogel-based sensor exhibited excellent mechanical resilience to pressure, showing potential application in the field of pressure sensors. Pressure sensitivity S is a key criterion revealing the sensitive ability of sensors, which is defined as S=δ(Δ*C*/*C*_0_)/δP in the present work, where Δ*C* is the change of capacitance, *C*_0_ is the initial capacitance without exerted pressure, and *P* is the applied pressure. Figure 5f illustrates that the capacitance of the C_11_T_4_A_15_ hydrogel gradually increased with increasing applied pressure, which exhibited a multistage sensitivity behavior. The pressure sensitivity (S) is 0.27, 0.13, and 0.06 kPa^−1^ when the applied pressure is within the ranges of 0–47, 47–180, and 180–600 kPa, respectively. In particular, the high soft C_11_T_4_A_15_ sensor showed S of 167.24 and 85.88 kPa^−1^ for very small pressure in the range of 0–0.03 and 0.03–0.13 kPa, respectively (Fig. S24). The pressure sensor combines the advantages of high sensitivity at low pressures and a wide response range, which is superior to some reported gel-based pressure sensors (Fig. S25).

Moreover, the sensor can detect ultra-small pressure down to 60 Pa and can discern a very small pressure change of 50 Pa (Fig. 5g), which further demonstrates the good pressure sensitivity of the C_11_T_4_A_15_ sensor. In addition, the sensor showed a fast response speed of 200 ms to applied pressure (Fig. S26). The long-term electrical stability and reproducibility of the C_11_T_4_A_15_ sensor were evaluated under cyclic compressive tests at a strain of 50% for 2000 times (Fig. S27). The capacitance change ratio remained constant under repeated loadings, demonstrating its remarkable electrical stability and durability when employed in practical applications.

### Real-Life Demonstration of Wearable Strain Sensors

Based on the superior mechanical properties, good conductivity, strong adhesive ability, and excellent sensing sensitivity of the CTA hydrogel, the wearable strain sensor was fabricated to demonstrate the potential application in biomonitoring, even the subtle movement of the muscles. For demonstration, the C_11_T_4_A_20_ hydrogel-based sensor was placed on different parts of the body for real-time motion monitoring and multiple physiological signal detection. As shown in Fig. 5h, our C_11_T_4_A_20_ sensor was pasted to the wrist for real-time monitoring artery pulse waveforms. Pulse is a significant physiological signal for systolic and diastolic blood pressure as well as heart rate. By monitoring a person’s pulse and collecting pulse waves, it is distinctly important for the diagnosis and prophylaxis of certain diseases. It distinctly exhibited repetitive and regular pulse shapes when the human body was at rest, in which there were 14 peaks in 12 s (70 pulses per min). As depicted in the inset of Fig. 5h, the photograph and enlarged diagram of a single pulse peak in relaxation clearly distinguish the characteristics of the pulse waveform, namely “P” (percussion), “T” (tidal), and “D” (diastolic) [60], demonstrating the high sensitivity of C_11_T_4_A_20_ sensor.

As shown in Fig. S28, the C_11_T_4_A_20_ sensor is pasted on the neck position to recognize the movement of the tiny epidermis and throat muscles during eating and drinking. The measured relative resistance variation includes three characteristic peaks, which is consistent with the theoretical change in resistance of the swallowing action [11, 61]. The sensor can accurately distinguish vibration signals between two vocal cords. When speaking English words, such as “Hydrogel”, “Welcome”, and “Thanks”, distinguishable and reproducible signal patterns are yielded, exhibiting promising application for phonetic recognition (Fig. 5i). Besides, the C_11_T_4_A_20_ sensor can also be attached to the skin near the cheek (insets in Fig. S29a) and eyebrow (insets in Fig. S29b) to detect subtle muscle movements induced by facial expressions. The relative resistance changes versus time for the stretching of facial muscle can accurately record, induced by facial expression changes from a smile face to a laughing face (Fig. S29a), from raising eyebrows to frown (Fig. S29b). Respiration rate and depth were monitored by attaching the C_11_T_4_A_20_ sensor on the abdomen. Figure S30 shows the respiration cycles consisting of three different modes of shallow breathing, fast breathing, and deep breathing.

Besides the softness that allowed arbitrary deformations for subtle strain sensing, the C_11_T_4_A_20_ sensor also possesses high mechanical adaptability to discriminate different large-range human motions, such as movements of finger, wrist, elbow, and knee joints. As depicted in Fig. 5j, the C_11_T_4_A_20_ sensor was pasted on the finger joint to monitor the movement of the finger. A stepwise increase/decrease in relative resistance was observed due to the bending/releasing of the finger. The relative resistance showed high stability while maintaining the bending angle of the forefinger, and can maintain a similar value in the process of bending and straightening of the forefinger at the same angle, which displayed the instantaneous and accurate measurement of finger movement. Similarly, when the sensor was attached to the wrist or elbow, the signals could clearly distinguish the bending of the wrist (Fig. 5k), and the bending amplitude of the elbow (Fig. S31), respectively. Moreover, we attached the C_11_T_4_A_20_ sensor on the knee joint to detect and discriminate different motion conditions of the knee, such as standing, walking, running, and jumping (Fig. S32). It can be clearly observed that the magnitude increased due to the large deformation caused by the movement. Based on the above results and the remarkable sensitivity and extremely wide sensing range of the wearable strain sensors, our CTA hydrogel could be considered as a promising platform for applications of wearable smart sensors.

### Integration of CTA Sensors and Electronic Skin Application

**Fig. 6:**
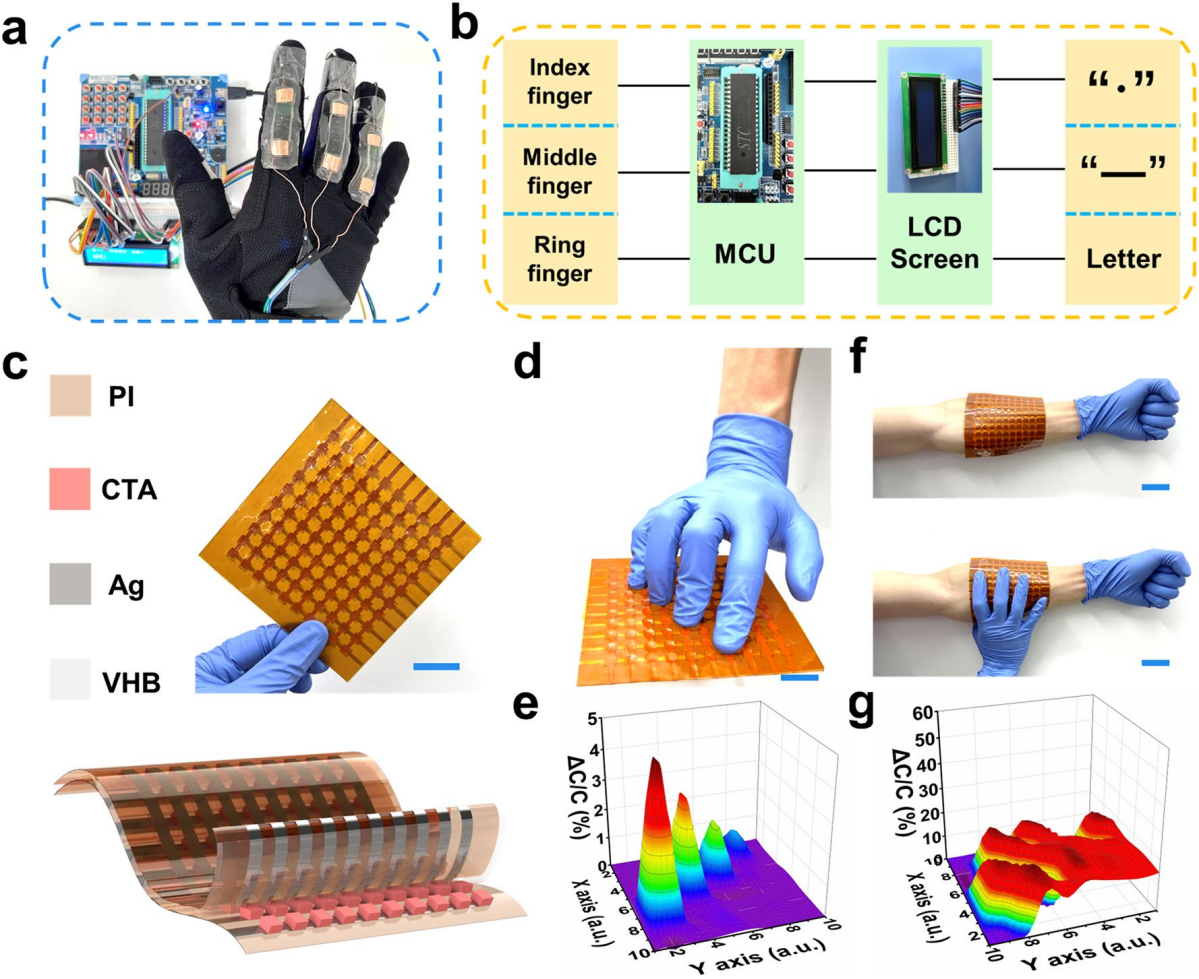
**a** Photograph of the human–machine interactive device based on the C_11_T_4_A_20_ sensors. **b** Schematic diagram of signal management circuit. **c** The integrated C_11_T_4_A_15_ hydrogel-based array electronic skin. The top right inset is a photograph of the integrated device. **d** Photograph of the electronic skin with four fingers touching. **e** Corresponding signal map showing the precise pressure distribution in **d**. **f** Photograph of the wearable electronic skin on an arm. **g** Corresponding signal map showing the precise pressure distribution in **f**. Scale bar: 4 cm

A flexible human–machine interactive system was developed based on the C_11_T_4_A_20_ sensors. The sensors were attached on fingers of a hand with VHB elastomers that come into contact with the glove (Fig. 6a). According to the working mechanism of strain sensor, a resistance signal generates during finger bending and straightening. If we define the occurring of resistance signal of index finger as “·”, the signal of middle finger as “-”, and the signal of ring finger as “Enter”, we can output letters according to Morse code (Table S4). A circuit diagram of the signal evolution in the human–machine interactive system is illustrated in Fig. 6b, which consists of wearable typewriter based on the C_11_T_4_A_20_ sensors to acquire the analog signals, the signals are converted into the corresponding value through a signal analysis and control unit (STC89C516RD +), and the processed digital signals are sent to LCD screen for human–machine interaction. The analog signals generated by wearable typewriter based on the C_11_T_4_A_20_ sensors require to be processed in signal processing circuit, and appropriate threshold voltage need be set to filter out noise and crosstalk signals. The abbreviation “WHU” of Wuhan University was written by bending and straightening the fingers as shown in Video S2. For example, the “W” was input by straightening the index finger, middle finger, middle finger, and ring finger in turn.

In addition, we demonstrate its application in the field of tactile sensing. It can be used as a tactile switch. Figure S33 and Video S3 show that the flexible tactile switch controls a digital thermometer and hygrometer by sensing the press and departure of the finger. Furthermore, for electronic skin applications, it is desirable to assemble the sensor into an array configuration with multiple pixels to collect spatially resolved pressure information. As a demonstration, we fabricated a 10 × 10 pixel array with a total area of 144 cm^2^, which composed of 100 C_11_T_4_A_15_ hydrogel-based press sensor units (0.5 × 0.5 cm^2^ per unit) assembled in the middle VHB encapsulate layer (Fig. 6c). The top and the bottom electrodes were fabricated via a printing method using silver paste on flexible polyimide (PI) substrates (50 μm in thickness). Figure S34a shows the response of the integrated array sensor to the placement of three weights with different weights. The 3D signal mapping corresponds well to the locations of the weights as shown in Fig. S34b. When fingers were placed on the integrated array sensor (Fig. 6d), the touch of the fingertips causes a local circular “point” pressure. A 3D mapping is output through gathering relative capacitance changes in the array (Fig. 6e), which graphically represents the spatial pressure distribution. More importantly, to further demonstrate the potential applications in skin-like electronics using the integrated sensor array on curved surfaces, we pasted the pressure sensor array around a volunteer’s forearm with one pressed hand (Fig. 6f). The capacitance mapping of the palm positions in Fig. 6f is shown in Fig. 6g. The areas with obvious variation in capacitance correspond well to the locations of the pressing palm.

### Electrical Output Performance of CTA Hydrogel-Based Stretchable Triboelectric Nanogenerator

**Fig. 7:**
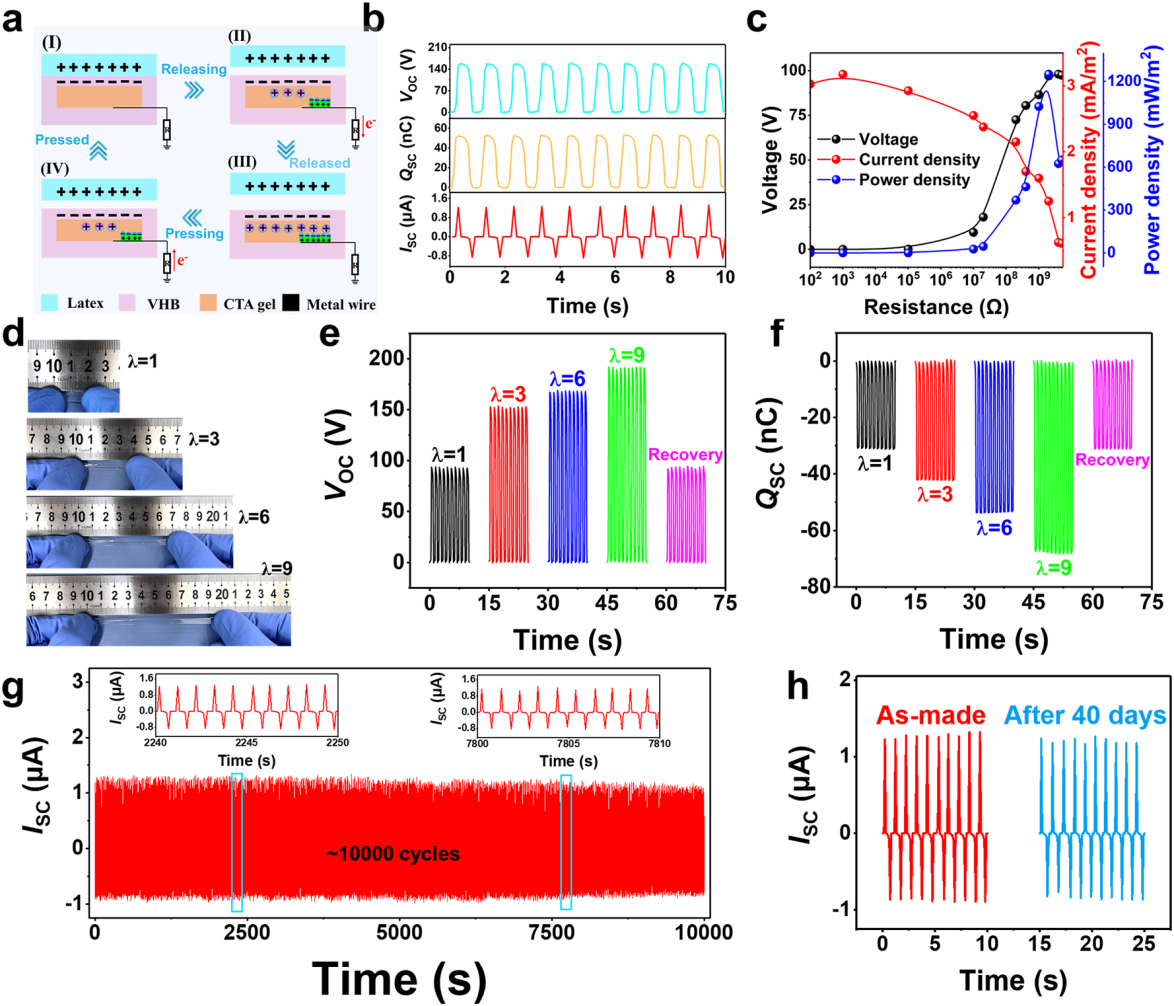
**a** Schematic exhibiting the working principle of CTA-TENG. **b** The corresponding *V*_OC_, *I*_SC_, and *Q*_SC_ of CTA-TENG. **c** Variation of the electrical output performances of CTA-TENG as a function of external resistors. **d** Photographs of a CTA-TENG in initial state and different stretched states. **e** *V*_OC_ of a CTA-TENG in initial state and different stretched states. **f** *Q*_SC_ of a CTA-TENG in initial state and different stretched states. **g** Long-term stability test of CTA-TENG. **h** *I*_SC_ of CTA-TENG after storage for 40 days

Owing to the ultrahigh mechanical stretchability and high conductivity, the CTA could be employed as the electrode layer to fabricate the stretchable TENG for biomechanical energy harvesting [62–64]. The CTA as work electrode was encapsulated by a 3 M VHB, which is usually used as both encapsulation and triboelectric material because of its high electronegativity, excellent elasticity, and optical transparency. A CTA hydrogel-based TENG (CTA-TENG) with a sandwich structure was constructed according to our previous report [5], including the commercial VHB as the contact triboelectric material layer, the C_11_T_4_A_20_ hydrogel as the electrode layer, and the copper connecting wire attached to the hydrogel for electrical connection. The stress–strain curve of CTA-TENG was measured, as shown in Fig. S35. The fabricated CTA-TENG works in a single-electrode mode based on the coupling between triboelectrification and electrostatic induction, and the detailed working principle of the CTA-TENG is schematically illustrated in Fig. 7a. Briefly, when the latex layer contacts with the VHB layer, electrification occurs at the interface and generates the same amount of charges with opposite polarities at the surface of the latex layer and the VHB layer, respectively (Fig. 7a(I)). There is practically no electrical potential difference between the two surfaces since the two opposite charges coincide at almost the same plane. When the two surfaces separate and move away, the static charges on the surface of the insulating VHB film will induce the movement of the ions in the C_11_T_4_A_20_ hydrogel to balance the static charges, forming a layer of excessive ions at the interface (Fig. 7a(II)). Then, the unscreened negative charge on the surface of VHB induces the accumulation of cationic ions at the VHB/C_11_T_4_A_20_ hydrogel interface and anionic ions at the C_11_T_4_A_20_ hydrogel/copper interface, which in turn leads to electrical double-layer formation [65, 66]. Meanwhile, a transient charge transfer flows from the copper connecting wire to the ground through the external circuit, generating an electrical signal. When latex layer and VHB layer are completely separated, an electrostatic equilibrium is formed at the interface between VHB layer and the C_11_T_4_A_20_ hydrogel and no electrical signal would be generated (Fig. 7a(III)). If the moving the latex layer is approaching back to the VHB layer, the whole process will be reversed and the electrons will transfer from the ground to the copper connecting wire through the external circuit, generating an electrical signal in the opposite direction (Fig. 7a(IV)). By repeating the contact-separation movement, an alternative current will be generated.

The typical electrical output measurements of the standard CTA-TENG (the size of 20 × 30 mm^2^ and the frequency of 1 Hz) were measured by a stepping motor to simulate the latex layer touching on VHB layer. As shown in Fig. 7b, it can be observed that the open-circuit voltage (*V*_OC_) of 158 V, the charge transfer (*Q*_SC_) amount of 52 nC, and the short-circuit current (*I*_SC_) peak value of 1.2 μA were achieved, respectively. To further evaluate the energy harvesting ability of the CTA-TENG in the external circuit, the effect of different external loads of 100 Ω-5 GΩ on the output voltage, the current density, and the instantaneous power density have been measured (Fig. 7c). Following the Ohm’s law [67], the output voltage of the CTA-TENG increased, while the current decreased when the connected load resistances increased. Moreover, the maximum peak value of the output power density (*P*_max_) was calculated to be 1.17 W m^−2^ at an external loading resistance of 1.5 GΩ. The obtained output results of the CTA-TENG were among the top class compared to that of previously reported TENG devices (Table S5). One major advantage of the C_11_T_4_A_20_ hydrogel is the ultrahigh stretchability, and the energy harvesting of the CTA-TENG in stretched states was further evaluated. A CTA-TENG (10 × 30 mm^2^) was uniaxially stretched for different stretches or strains (Fig. 7d), and corresponding electrical outputs were recorded under contact-separation motion (Fig. 7e, f). Compared with the initial state without strain, the *V*_OC_ of the CTA-TENG was continuously improved from ~ 93 to ~ 152 and ~ 192 V after being stretched for λ = 3 and 9, respectively, due to the increase in the surface area of the VHB layer and thus the contacting area for the electrification at stretched states [68]. The *Q*_SC_ of the CTA-TENG follows the similar trend when the contacting area increases. After recovering from the stretched states, the electrical output is comparable with the initial state, suggesting no degradation of the device. The long-term electrical output stability of the CTA-TENG was further examined by performing contact-separation motion over 10,000 times at a frequency of 1 Hz, as shown in Figs. 7g and S36, which exhibits the superior stability of electrical output under long-term testing and reveals the mechanical durability of the CTA-TENG. Meanwhile, the durability and stability of the CTA-TENG were also evaluated after storage for 40 days in ambient environment. No significant degradation was observed (Figs. 7h and S37) for the electrical output, demonstrating the favorable anti-drying ability of the CTA-TENG and satisfying the requirements of the reliability for a practical nanogenerator.

## Conclusions

In summary, a class of physically crosslinked CTA hydrogels were successfully developed through facile heated polymerization of AAm in the presence of CECT. The abundant noncovalent interactions and vigoroso crosslinked network structure endowed the CTA with excellent mechanical performance (strain up to 1586%, toughness up to 1300 kJ m^−3^), strong self-adhesive properties (113 kPa for pigskin), high ionic conductivity (0.62 S m^−1^), and remarkable optical transparency (92%). Taking advantage of these features, high-performance stretchable wearable strain sensors with high sensitivity (GF up to 18.54), low limit of detection (0.1% strain), timely response (61 ms), and excellent reliability and durability (up to 2000 cycles) have been constructed for the detection of not only large body motions (bending of fingers and limbs) but also delicate and complex small muscle movements (pulsing, speaking, frowning, and swallowing) and the real-time monitoring of human motion with quantification. The CTA-based pressure sensors also present outstanding sensing performances including ultrahigh sensitivity (167.24 kPa^−1^), wide pressure sensing range (0–600 kPa), and low detection limitation (60 Pa). Furthermore, the proof-of-concept demonstrations of the CTA, as soft and conformal bioelectronic devices, are carried out, including a human–machine interactive system and electronic skin for unprecedented nonplanar pressure sensing. As a self-charging power system, the single-electrode stretchable CTA-TENG exhibited favorable energy harvesting performance with reliable stability and satisfied deformation conditions (300%–900% strain). Considering the simple preparation, integration of multiple functions, and excellent comprehensive performances, it is believed that this study would provide insights for developing high-performance hydrogels for next-generation wearable electronics, soft robotics, HMI, big data, and other fields.

### Supplementary Information

Below is the link to the electronic supplementary material.

Supplementary file1 (MP4 6713 kb)

Supplementary file2 (MP4 22720 kb)

Supplementary file3 (MP4 14860 kb)

Supplementary file4 (PDF 3089 kb)
