# Supplementary material for: Transparent, Ultra-Stretching, Tough, Adhesive Carboxyethyl Chitin/Polyacrylamide Hydrogel Toward High-Performance Soft Electronics
Source: Nanomicro Lett. 2022 Dec 7;15:8. doi: 10.1007/s40820-022-00980-9 (PMC9729505; doi:10.1007/s40820-022-00980-9)
Supplement: Supplementary file 4 — Supplementary file4 (PDF 3089 kb) [file 40820_2022_980_MOESM4_ESM.pdf]

Supporting Information for

Transparent, Ultra-Stretching, Tough, Adhesive Carboxyethyl Chitin/Polyacrylamide Hydrogel toward High-Performance Soft Electronics

Jipeng Zhang[1, 2], Yang Hu[1, 2], Lina Zhang[1, 2], Jinping Zhou[1, 2], and Ang Lu[1, 2,] *

[1] College of Chemistry and Molecular Sciences, Wuhan University, Wuhan 430072, People's Republic of China

[2] Hubei Engineering Center of Natural Polymer-based Medical Materials, Wuhan University, Wuhan 430072, People's Republic of China

*Corresponding author. E-mail: anglu@whu.edu.cn (Ang Lu)

S1 Supplementary Experimental Section

S1.1 Mechanical Property Test

Tensile and compressive measurements were tested on the hydrogels using a universal tensile-compressive tester (INSTRON instrument, Model 5576, USA). For tension, hydrogel membranes with a length of 50 mm, a width of 13 mm and a thickness of about 1.5 mm were measured at the speed of 50 mm·min^{-1}. For compression, columnar hydrogels with a height of about 10 mm and a diameter of 15.5 mm were tested at the speed of 2 mm min^{-1}. Young's modulus was calculated from the initial linear region of the stress-strain curves. The fracture energy (toughness) was calculated from full region of the stress-strain curves.

S1.2 Adhesion Performance Testing of Hydrogel Samples

The adhesion strength was determined by the lap-shear test using a universal test machine (INSTRON instrument, Model 5576, USA). The glass, plastic, wood, metal substrates and pig skin without contaminants were cut into rectangle with a length of 40 mm and a width of 15 mm. Hydrogel samples (10 × 10 × 1.5 mm^3) were sandwiched between two substrates with an area of 10 × 10 mm^2. After preloaded by 1kg weight for 10 min, the specimens were tested by the standard lap-shear test at a velocity of 10 mm min^{-1} under ambient conditions. The adhesion strength was calculated by dividing the maximum force by the adhesion area. Additionally, the adhesion-strip cyclic tests were also conducted to evaluate the effect of a cycle load on the adhesion strength of the hydrogels.

S1.3 Conductivity Assessment

Ionic conductivity of the hydrogels was measured by the electrochemical impedance spectroscopy (EIS) using an electrochemical workstation 165 (CHI760E, CH Instruments Ins) operated in the frequency range of 100 to 100 kHz and the amplitude of 5 mV. The hydrogels

were sandwiched between two carbon cloths for the measurement. The ionic conductivity (σ, S m^{-1}) of the hydrogels was calculated according to the following equation:

$$\sigma = \frac{L}{RS} \quad \text{(S1)}$$

where L (m), S (m^2), and R (Ω) was the length between two carbon cloths, the contact area of the hydrogel with carbon cloths, and resistance obtained by the intercept at the real part in Nyquist plots, respectively.

S1.4 Electrical Measurement

The electrical signals of the hydrogels were recorded by a capacitance meter (CAPACITANCE TESTER, UC2652, UCE Technologies). The change in the relative resistance/capacitance of the hydrogel sensors was examined using the above-mentioned capacitance meter at a constant voltage of 1 V, on the basis of different strains, and human motions. Relative changes in resistance and capacitance were calculated as the following equations:

$$\frac{\Delta R}{R_0} = \frac{R - R_0}{R_0} \times 100\% \quad \text{(S2)}$$

$$\frac{\Delta C}{C_0} = \frac{C - C_0}{C_0} \times 100\% \quad \text{(S3)}$$

where R_0, C_0 and R, C are the original resistance, capacitance at the strain of 0% at room temperature and the real-time resistance, capacitance at a certain strain, respectively.

S1.5 Characterization

^{1}H NMR spectra were recorded on a Bruker Avance-III 400 MHz spectrometer at room temperature. Field emission scanning electron microscopy (FESEM, Zeiss, SIGMA, Germany) was used to characterize morphologies of lyophilized hydrogels. Fourier transform infrared spectroscopy (FT-IR) of lyophilized hydrogels were tested by a Nicolet 170-SX (Thermo Nicolet Ltd., USA) in the wavenumber range from 4000 to 400 cm^{-1}. X-ray photoelectron spectra (XPS, ESCALAB250Xi, Thermo Fisher Scientific, America) analyses were recorded using a Kratos XSAM800 X-ray photoelectron spectrometer. Optical transmittance of the hydrogel films with a thickness of 1.5 mm was observed with a UV-vis spectrometer (UV-6, Shanghai Meipuda Instrument Co., Ltd., China) at a wavelength from 900 to 200 nm. Raman spectroscopy and spatial Raman mapping were performed using a Raman imaging microscope (Thermo Scientific DXR xi, USA). The wavelength of the excitation laser was 532 nm. The collected spectra were preprocessed using cosmic ray removal, noise filtering, and normalization techniques. The multivariate curve resolution (MCR) method developed by OMNICxi software was applied for calculating the proportion of interaction domains.

S2 Supplementary Videos

Video S1 (.mp4 format). Demonstration of the strong self-adhesive properties of the CTA hydrogel by vigorously swinging the hand.

Video S2 (.mp4 format). The proof-of-concept demonstrations of the CTA hydrogel as a human-machine interactive system.

Video S3 (.mp4 format). Demonstration of the CTA hydrogel in the field of tactile sensing as a tactile switch.

S3 Supplementary Figures

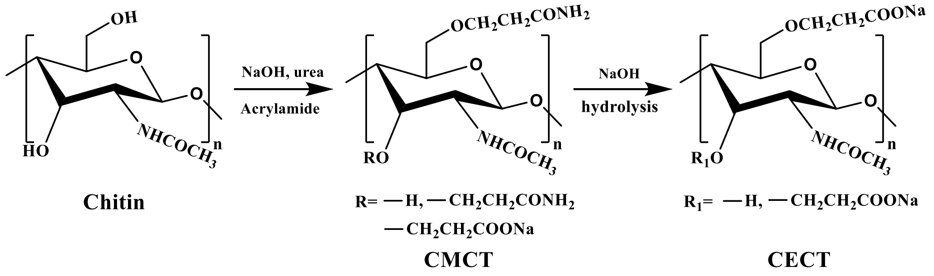

Scheme S1 Synthesis route of CECT

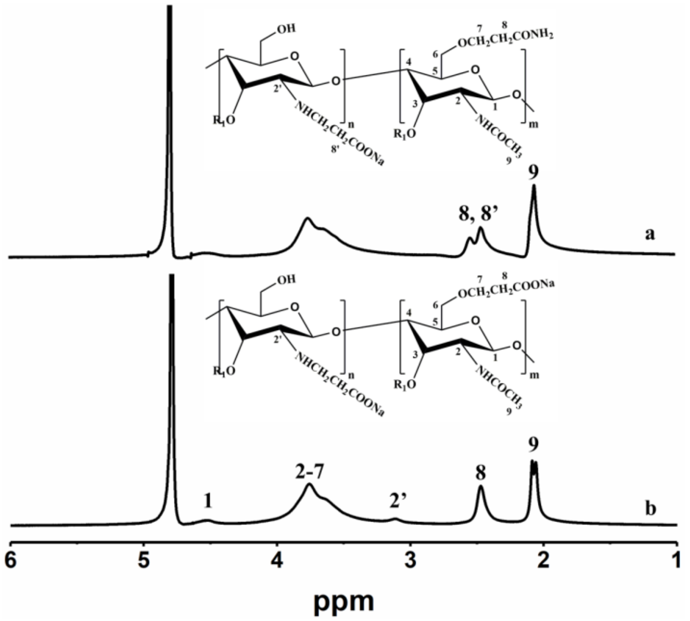

Fig. S1 ^{1}H NMR spectra of (**a**) CMCT, (**b**) CECT

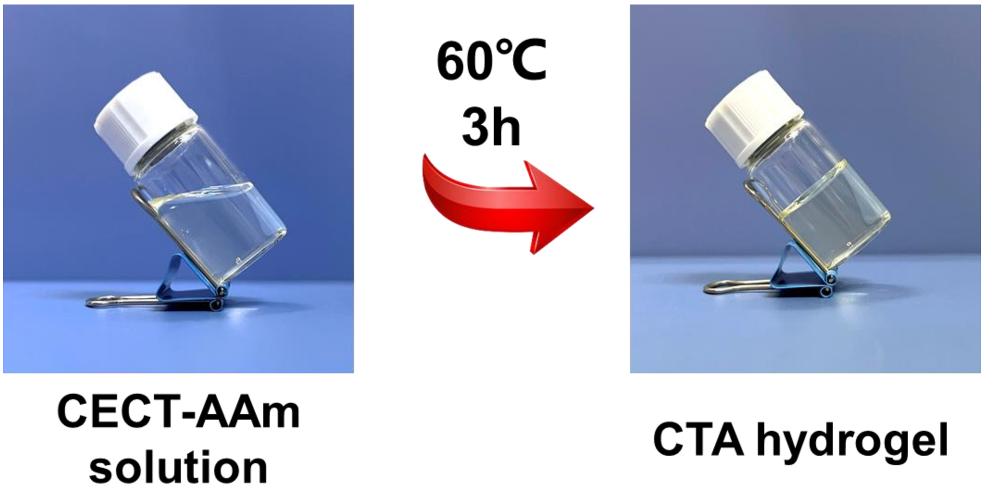

Fig. S2 Preparation of CTA hydrogel

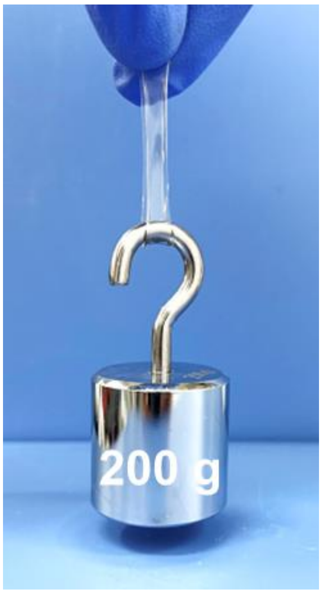

Fig. S3 Photographs of the stretching behavior for CTA hydrogel under a weight loading of 200

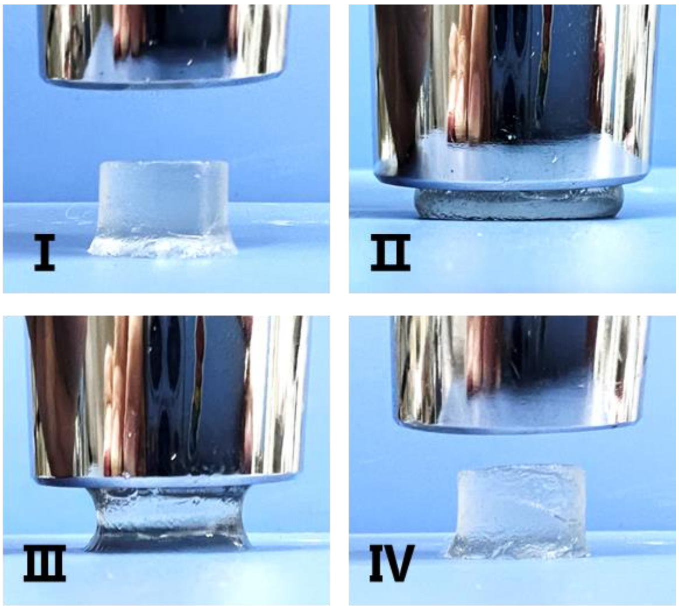

Fig. S4 Photographs of compressing the cylindrical CTA hydrogel with a heavy loading

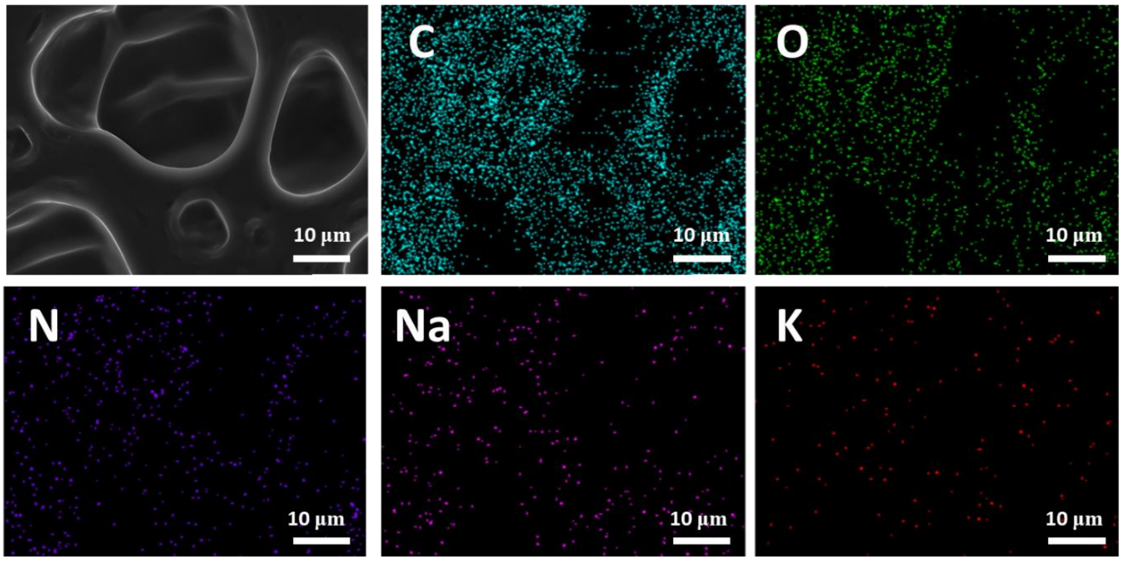

Fig. S5 Corresponding elemental mapping images of the CTA hydrogel

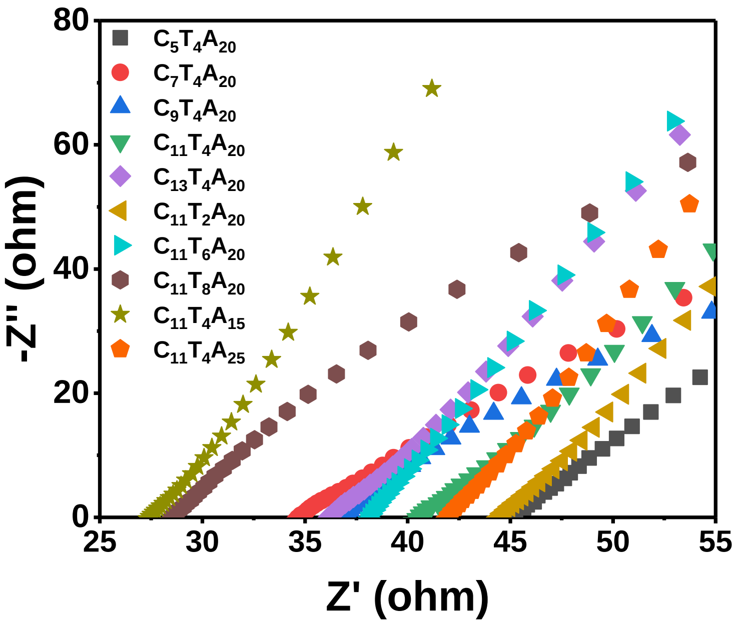

Fig. S6 EIS Nyquist plot of CTA hydrogel

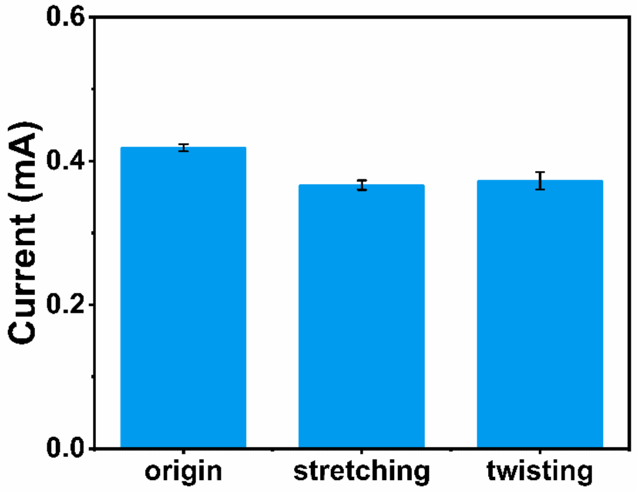

Fig. S7 Current values of $C_{11}T_4A_{20}$ hydrogel in different deformation states

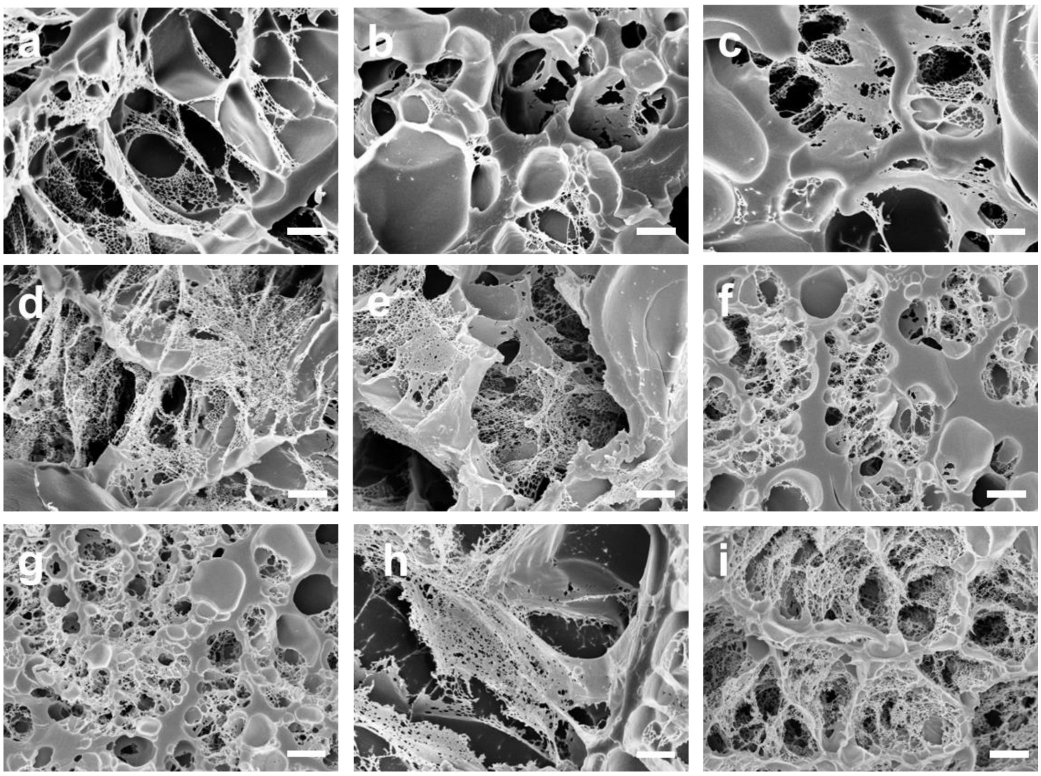

Fig. S8 SEM images of (**a**) $C_5T_4A_{20}$, (**b**) $C_7T_4A_{20}$, (**c**) $C_9T_4A_{20}$, (**d**) $C_{13}T_4A_{20}$, (**e**) $C_{11}T_2A_{20}$, (**f**) $C_{11}T_6A_{20}$, (**g**) $C_{11}T_8A_{20}$, (**h**) $C_{11}T_4A_{15}$ and (**i**) $C_{11}T_4A_{25}$ hydrogels. Scale bar: 2 μm

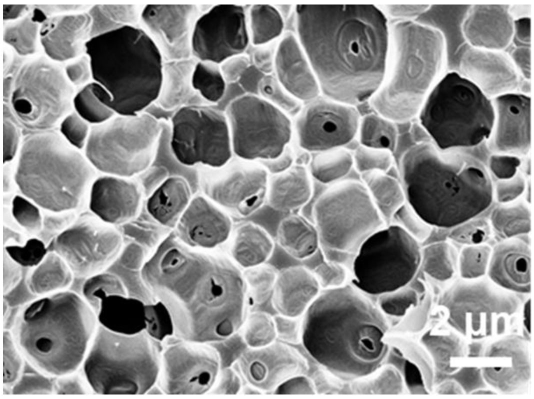

Fig. S9 SEM image of $C_0T_0A_{20}$ hydrogel

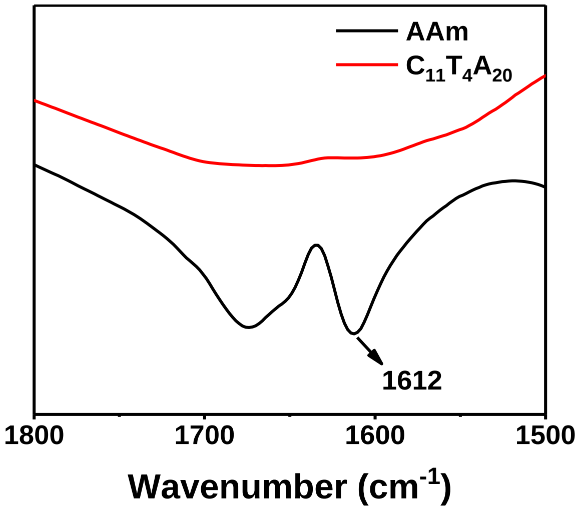

Fig. S10 FT-IR spectra of monomer AAm and $C_{11}T_4A_{20}$ hydrogel

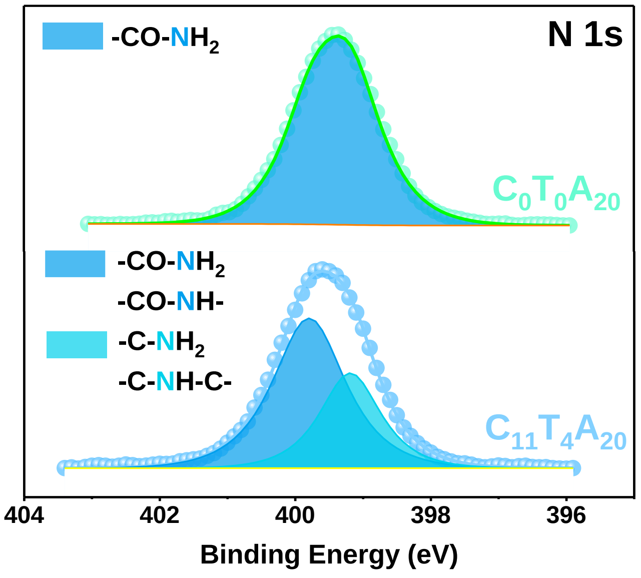

Fig. S11 N 1*s* XPS spectra for $C_0T_0A_{20}$ and $C_{11}T_4A_{20}$ hydrogel

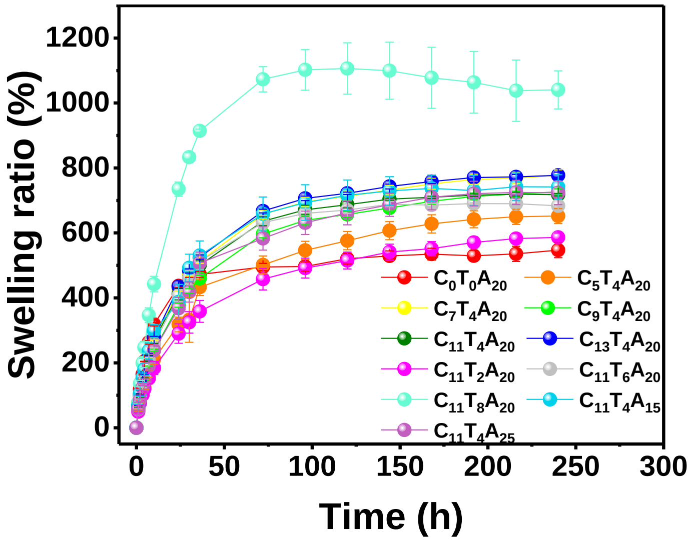

Fig. S12 Equilibrium swelling curves of CTA hydrogels in water

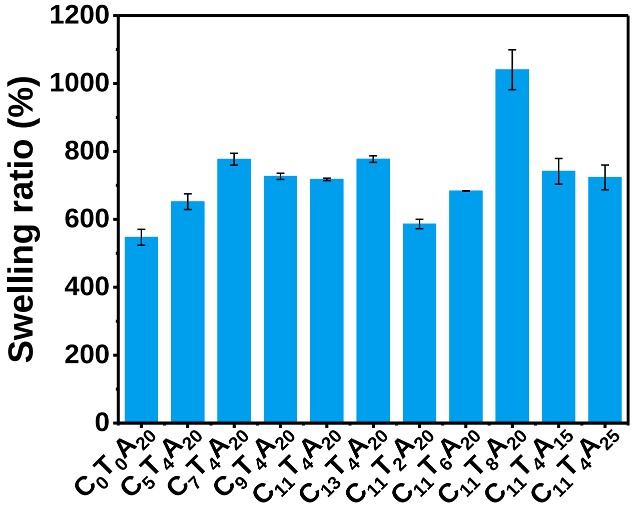

Fig. S13 Equilibrium swelling ratios of CTA hydrogels in water

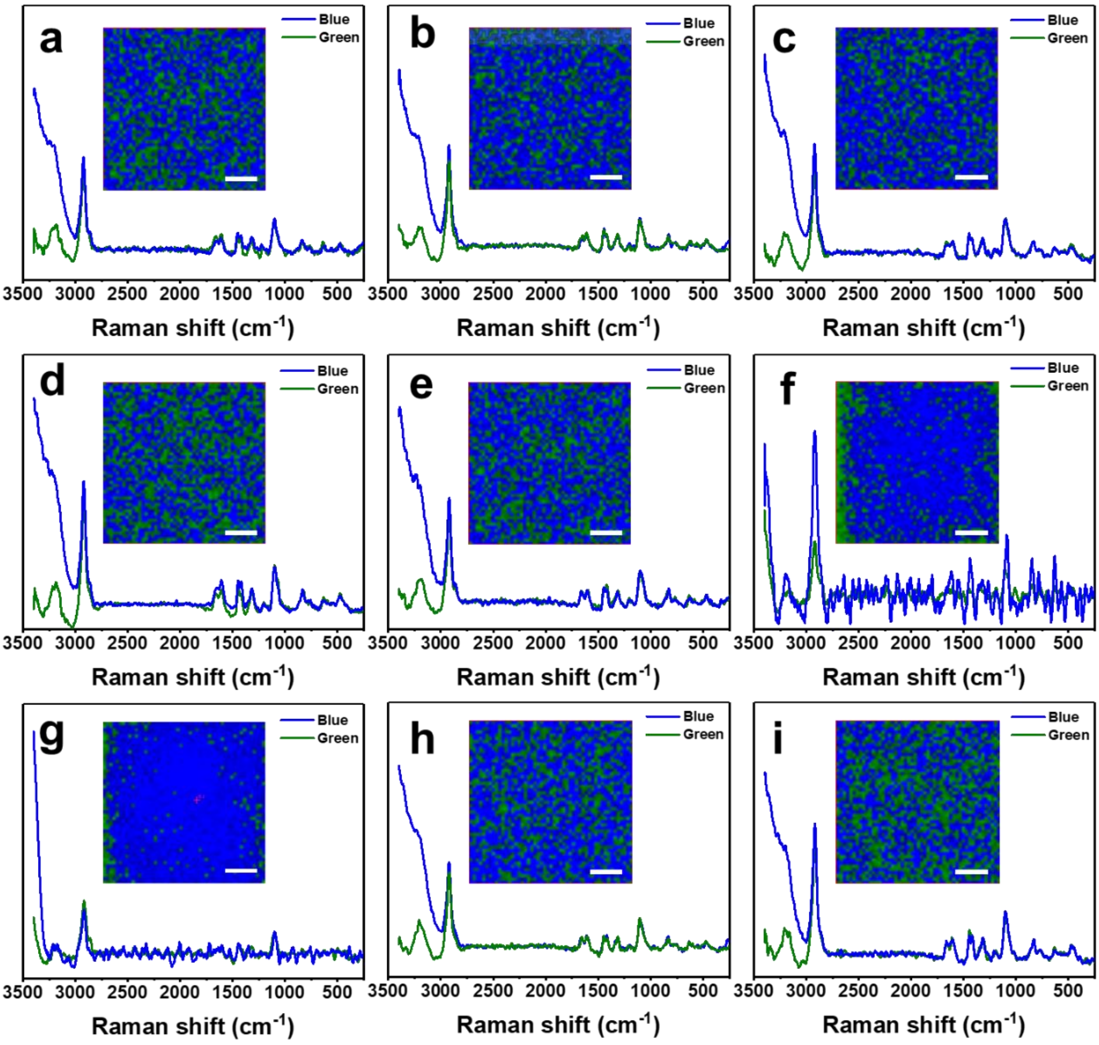

Fig. S14 Raman spectra of individual hydrophilic domains (blue) and hydrophobic domains (green) within (**a**) $C_5T_4A_{20}$, (**b**) $C_7T_4A_{20}$, (**c**) $C_9T_4A_{20}$, (**d**) $C_{13}T_4A_{20}$, (**e**) $C_{11}T_2A_{20}$, (**f**) $C_{11}T_6A_{20}$, (**g**) $C_{11}T_8A_{20}$, (**h**) $C_{11}T_4A_{15}$ and (**i**) $C_{11}T_4A_{25}$ hydrogels. The insets are the reconstructed MCR Raman mappings of hydrophilic domains (blue) and hydrophobic domains (green) obtained from -OH and -NH stretching mode intensities (3000-3400 cm^{-1}). All bars are 20 μm

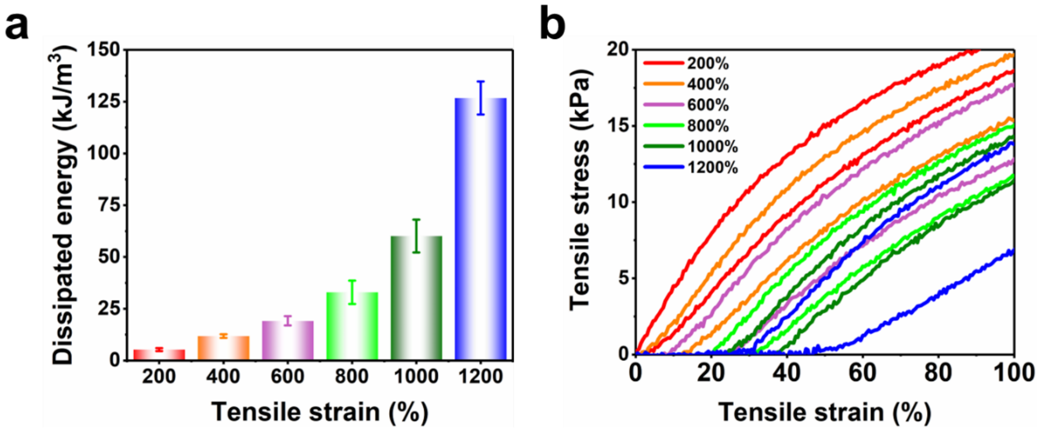

Fig. S15 (**a**) Dissipated energy of $C_{11}T_4A_{20}$ sample under various tensile strains. (**b**) Magnifying stress-strain curves between 0–100% for checking residual strain after stretching

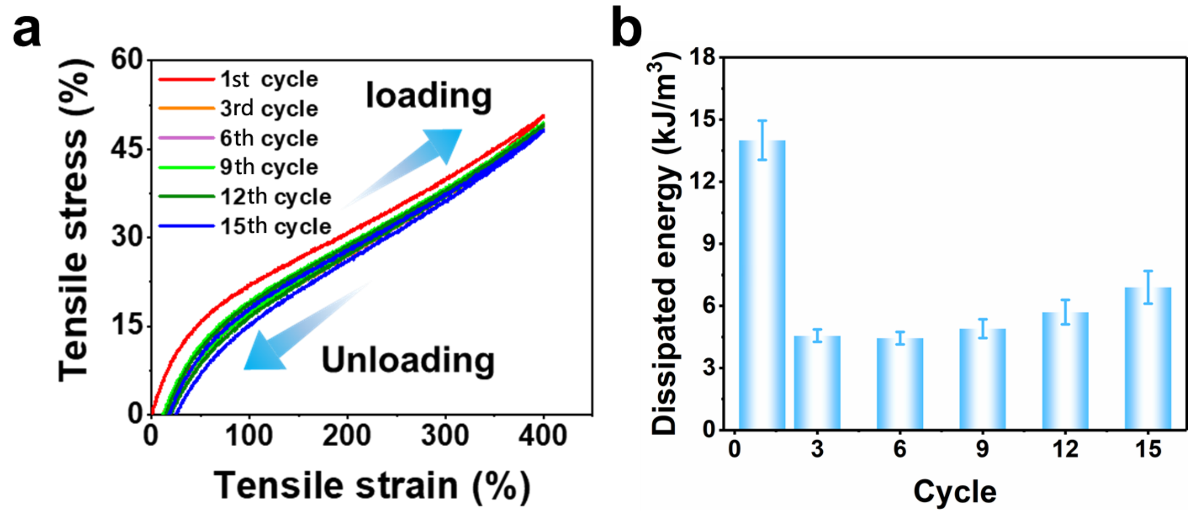

Fig. S16 (**a**) Successive loading–unloading curves of $C_{11}T_4A_{20}$ under 400% strain for 15 cycles. (**b**) Dissipated energy of $C_{11}T_4A_{20}$ sample under tensile loading-unloading cycles

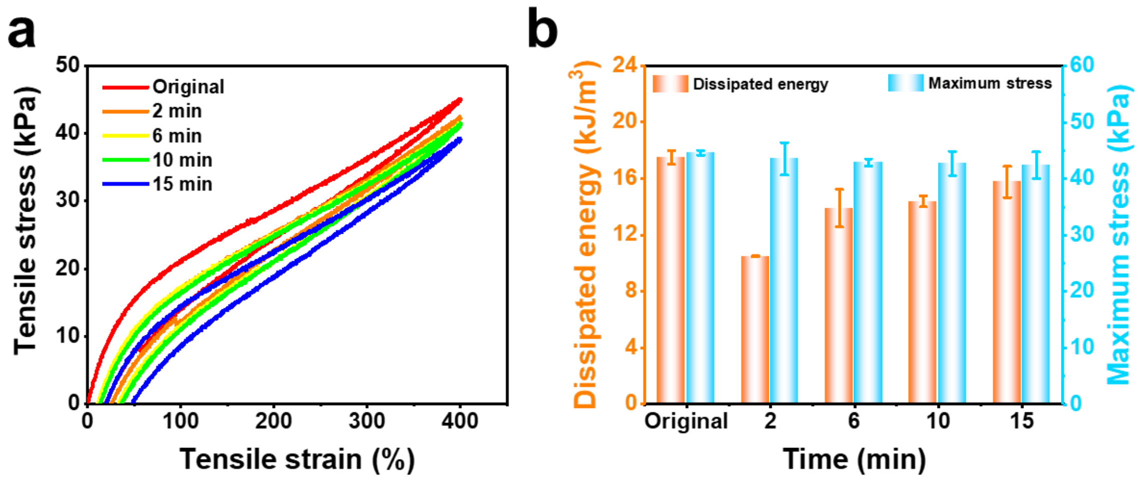

Fig. S17 (**a**) Tensile recovery test of $C_{11}T_4A_{20}$ sample with different recovery times. (**b**) Tensile dissipated energy and maximum stress during the tensile recovery test with different recovery times

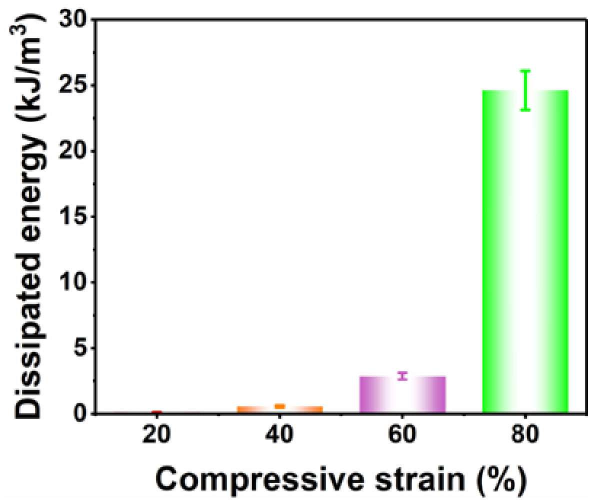

Fig. S18 Dissipated energy of $C_{11}T_4A_{15}$ sample under various compressive strains

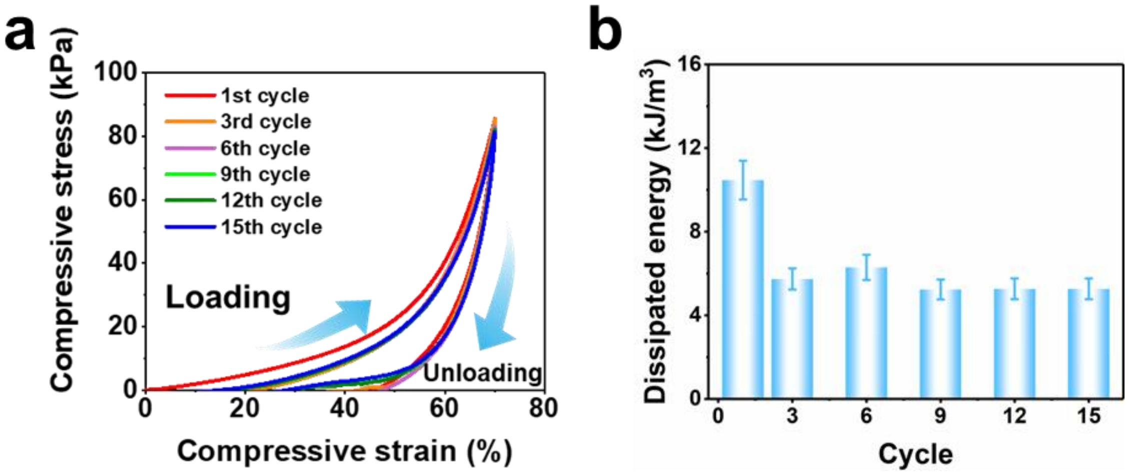

Fig. S19 (**a**) Successive loading−unloading curves of $C_{11}T_4A_{15}$ under 70% strain for 15 cycles. (**b**) Dissipated energy of $C_{11}T_4A_{15}$ sample under press loading-unloading cycles

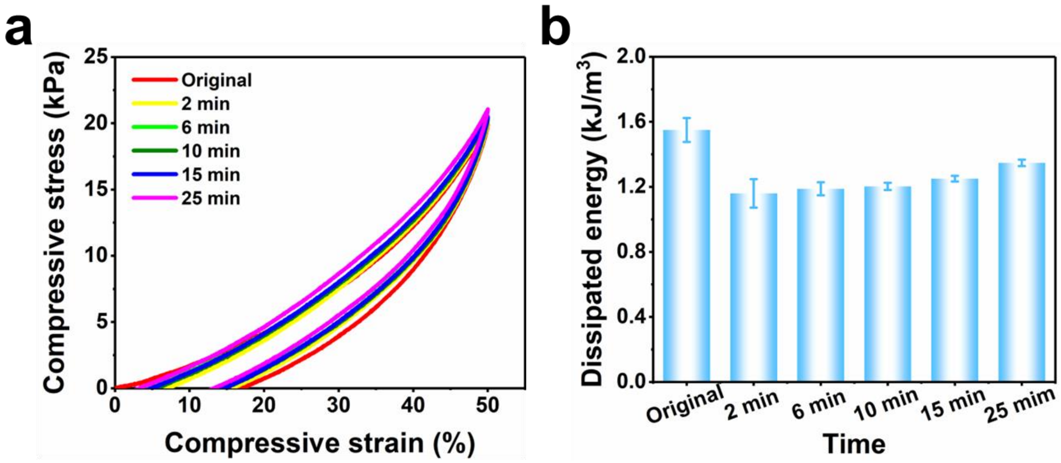

Fig. S20 (**a**) Press recovery test of $C_{11}T_4A_{15}$ sample with different recovery times. (**b**) Press dissipated energy during the press recovery test with different recovery times

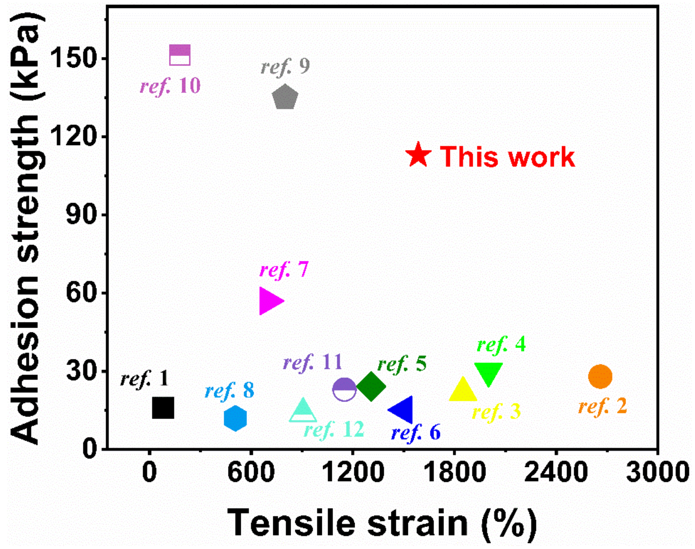

Fig. S21 Comparison of adhesion strength and tensile strain of reported conductive hydrogels [S1-S12]

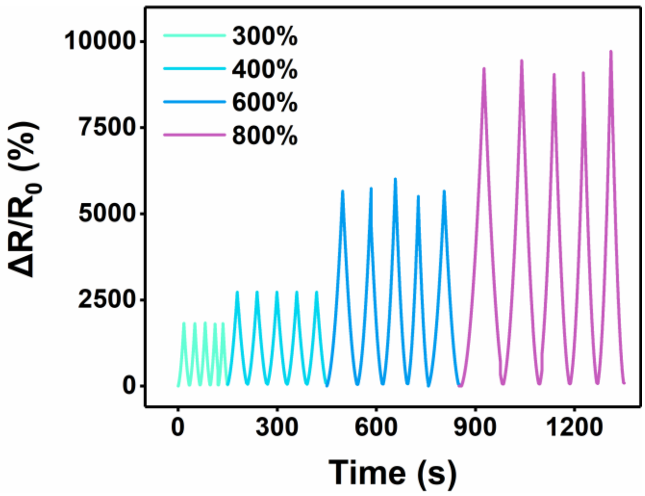

Fig. S22 Cyclic sensing behaviors of the $C_{11}T_4A_{20}$ hydrogel-based sensor upon stretching towards high strains ranging from 300% to 800%

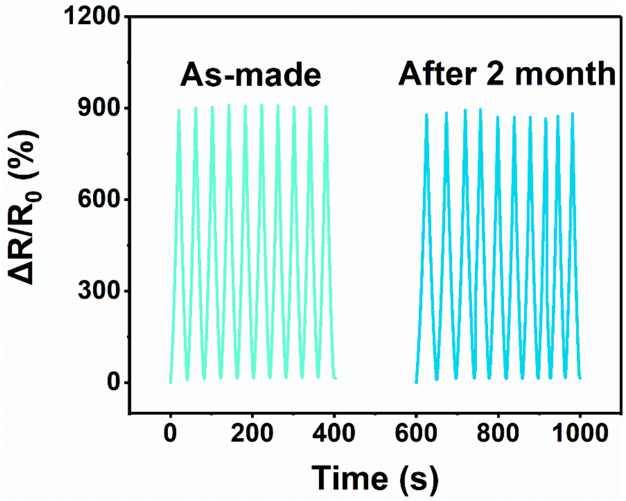

Fig. S23 Dynamic response of the encapsulated $C_{11}T_4A_{20}$ hydrogel-based sensor being placed in environment with 5 ºC, 48% humidity for 2 months to cyclic loadings

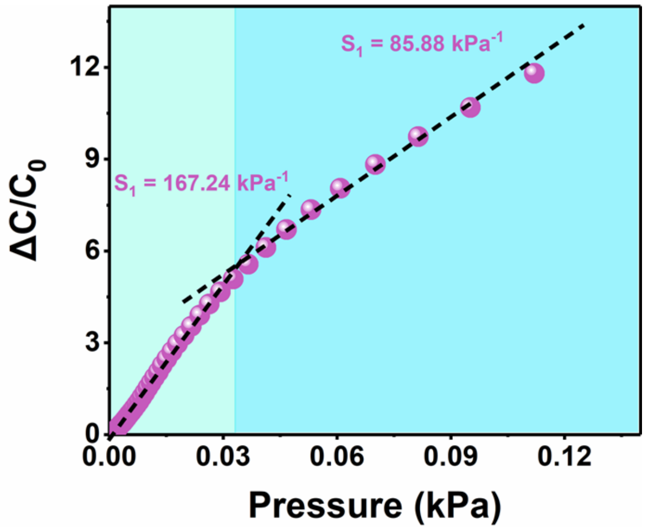

Fig. S24 Relative capacitance changes versus applied pressure within pressure range of 0–0.13 kPa of $C_{11}T_4A_{15}$

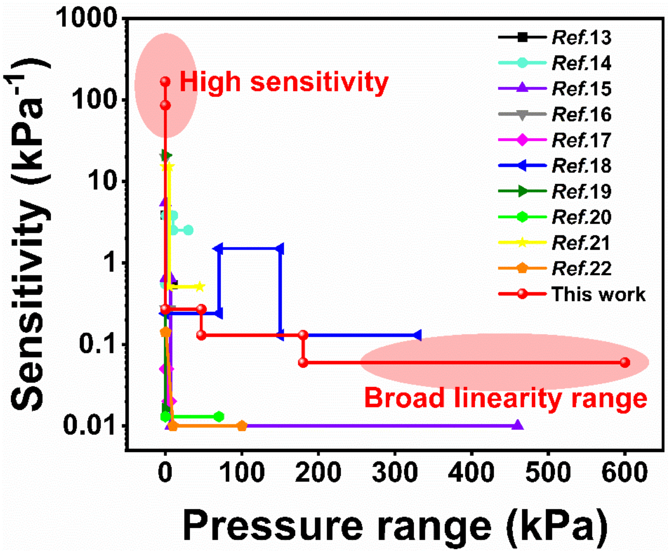

Fig. S25 Comparison of sensitivity and pressure range with literatures [S13-S22]

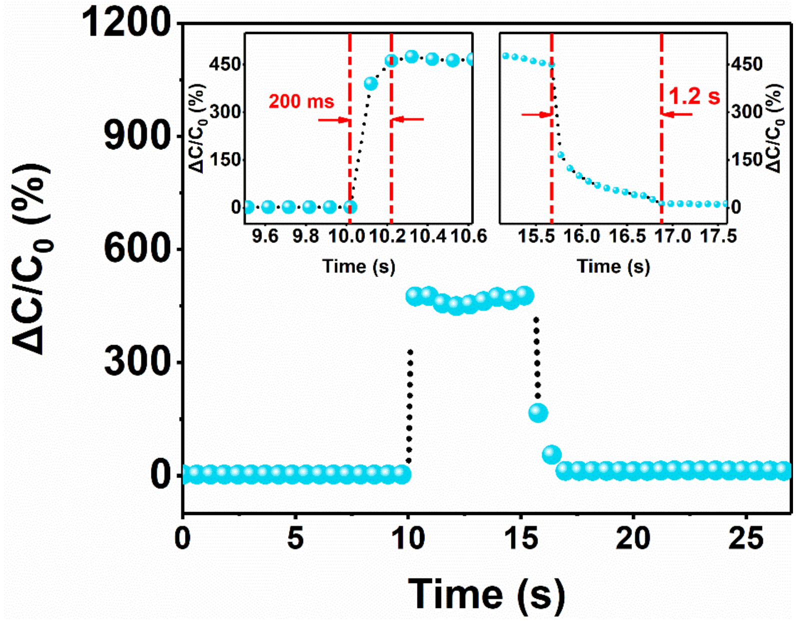

Fig. S26 The response time of the $C_{11}T_4A_{15}$ hydrogel-based sensor to the applied pressure

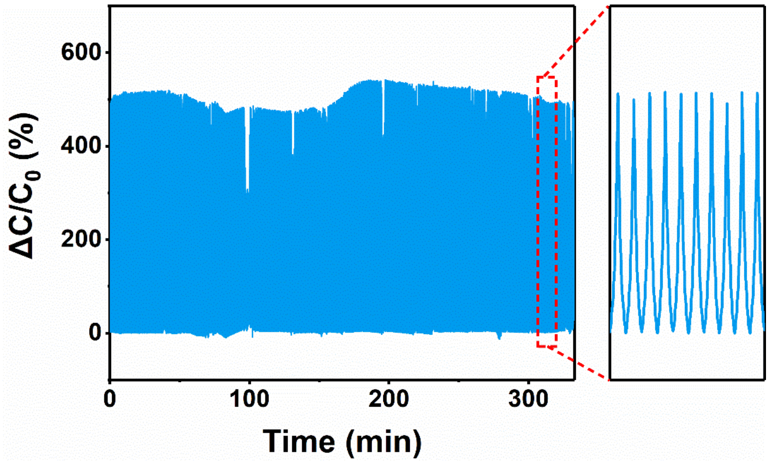

Fig. S27 Cyclic stability test of the $C_{11}T_4A_{15}$ hydrogel-based sensor under 50% compressive strain for 2000 cycles

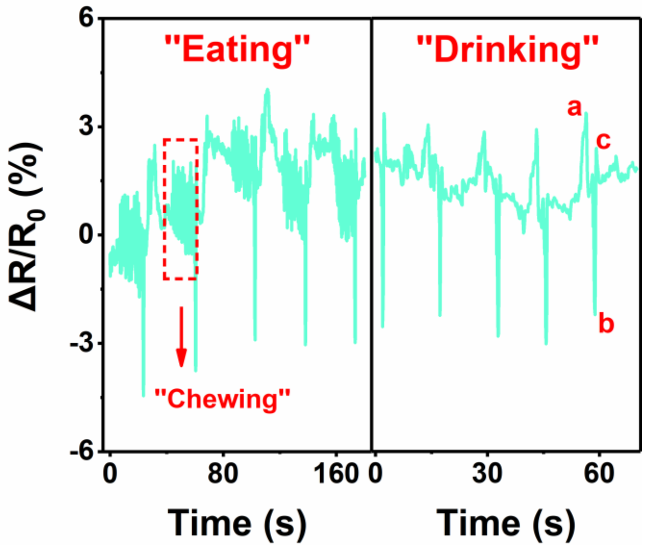

Fig. S28 Responsive signals of $C_{11}T_4A_{20}$ hydrogel-based sensor in monitoring tiny muscle movement of eating, chewing and drinking

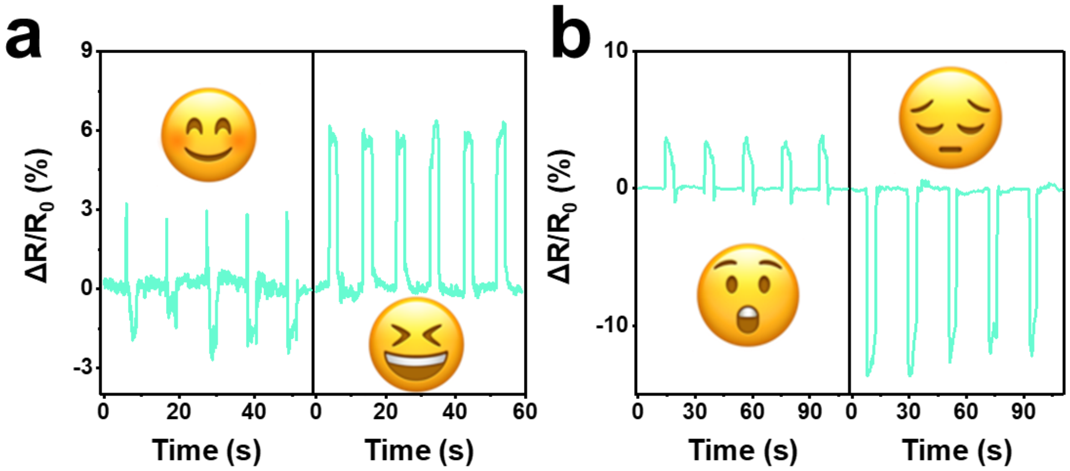

Fig. S29 Responsive signals of $C_{11}T_4A_{20}$ hydrogel-based sensor in monitoring tiny muscle movement of (**a**) smile, laughing and (**b**) raise, frown

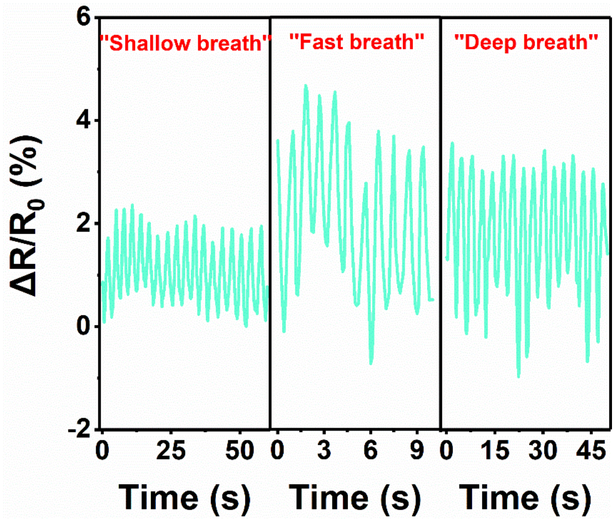

Fig. S30 Responsive signal of three different respiration modes: shallow breath, fast breath and deep breath

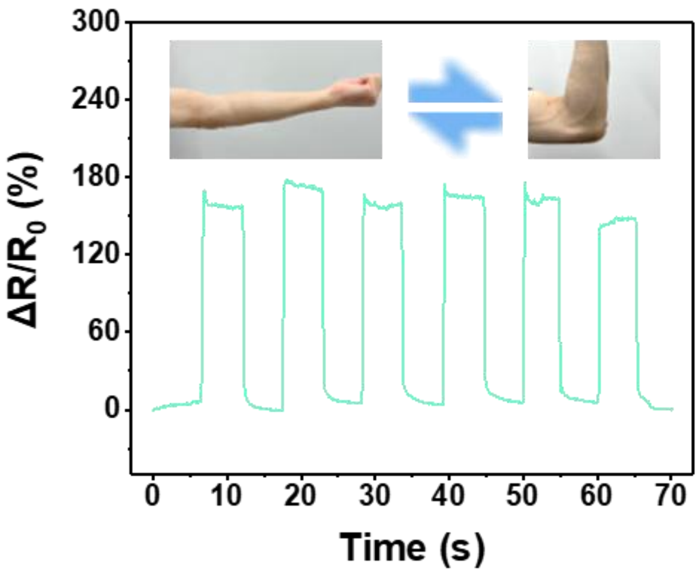

Fig. S31 Relative resistance changes with bending of the elbow

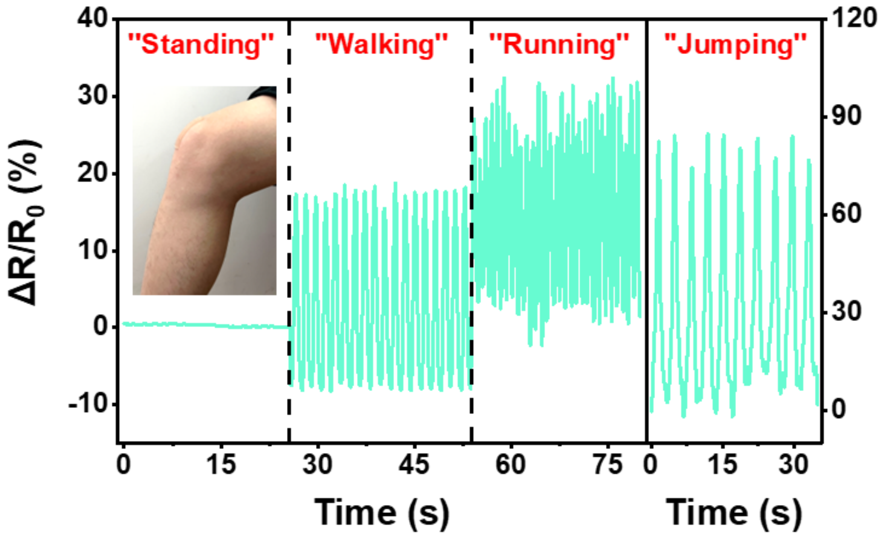

Fig. S32 Photograph of sensor attached on a knee joint, and corresponding signals of standing, walking, running, and jumping

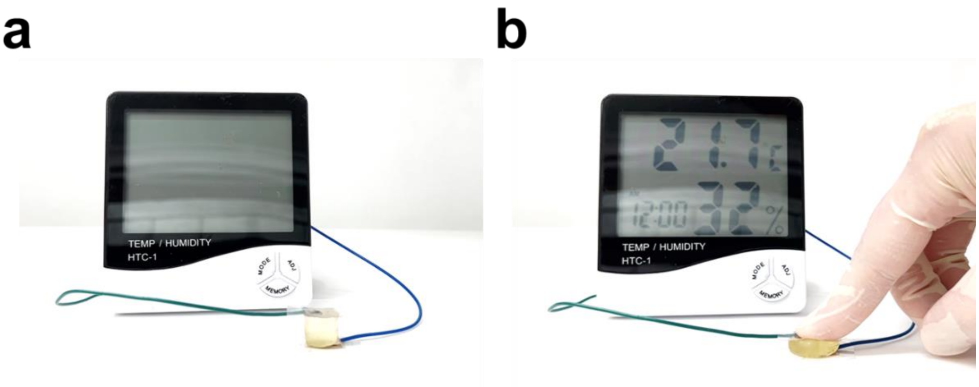

Fig. S33 (**a**) Photograph of a flexible tactile switch and a digital thermometer/hygrometer. (**b**) Photograph of a volunteer turning on the digital thermometer/hygrometer by pressing the flexible tactile switch

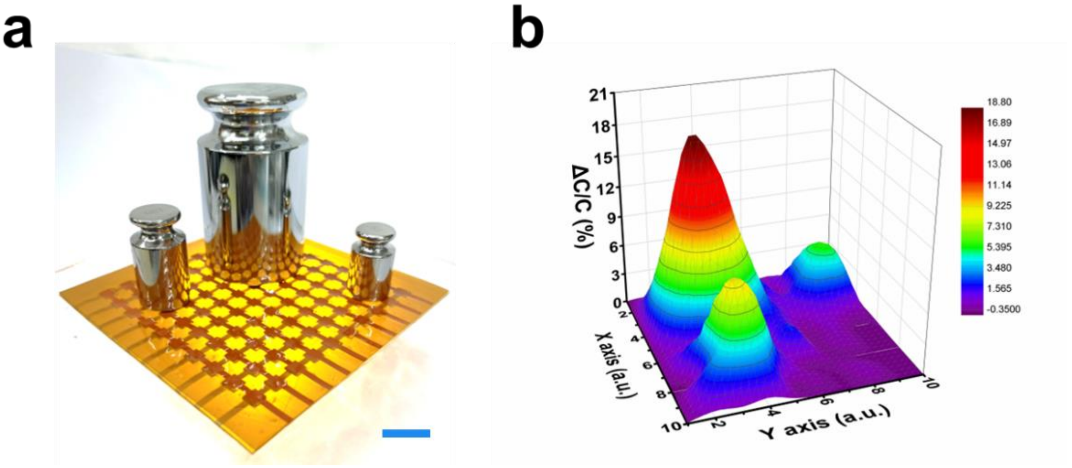

Fig. S34 (**a**) Photograph showing three weights distributed on the surface of an integrated 10 × 10 pressure sensor array (scale bar: 3 cm). (**b**) Corresponding signal map showing the precise pressure distribution in (**a**)

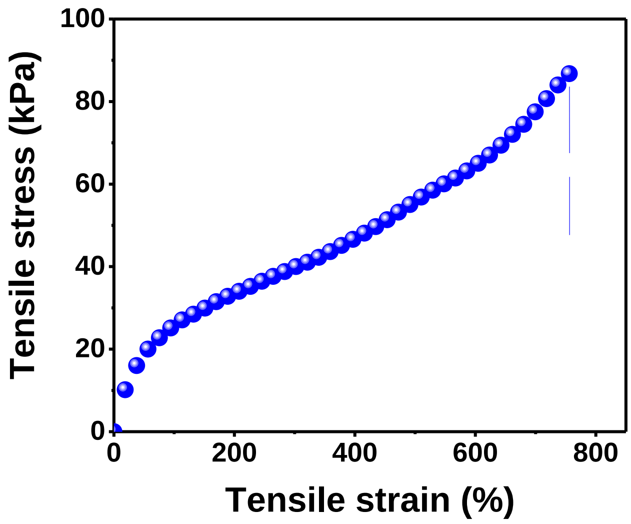

Fig. S35 The stress-strain curve of $C_{11}T_4A_{20}$-VHB based single-electrode CTA-TENG

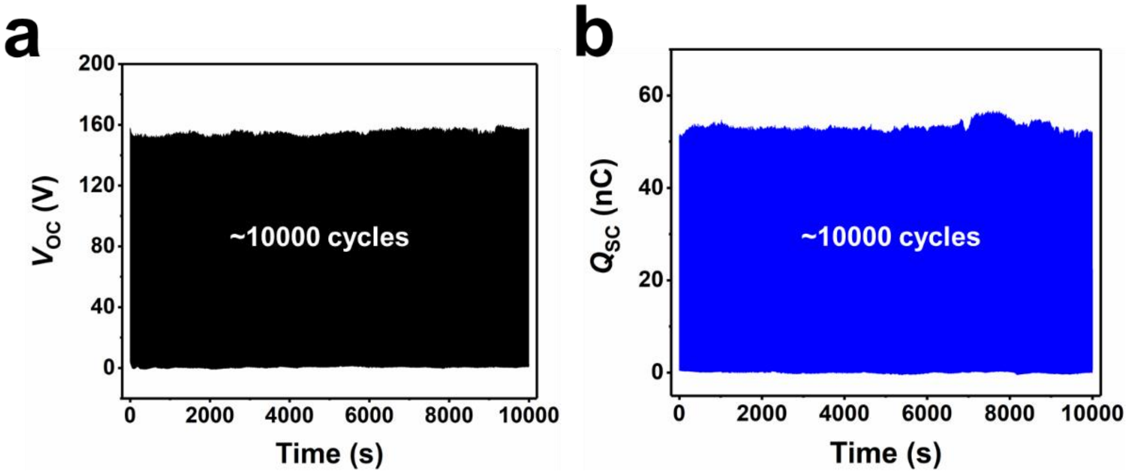

Fig. S36 V_{OC} and Q_{SC} of CTA-TENG that lasted for ~10000 cycles of contact-separation motions

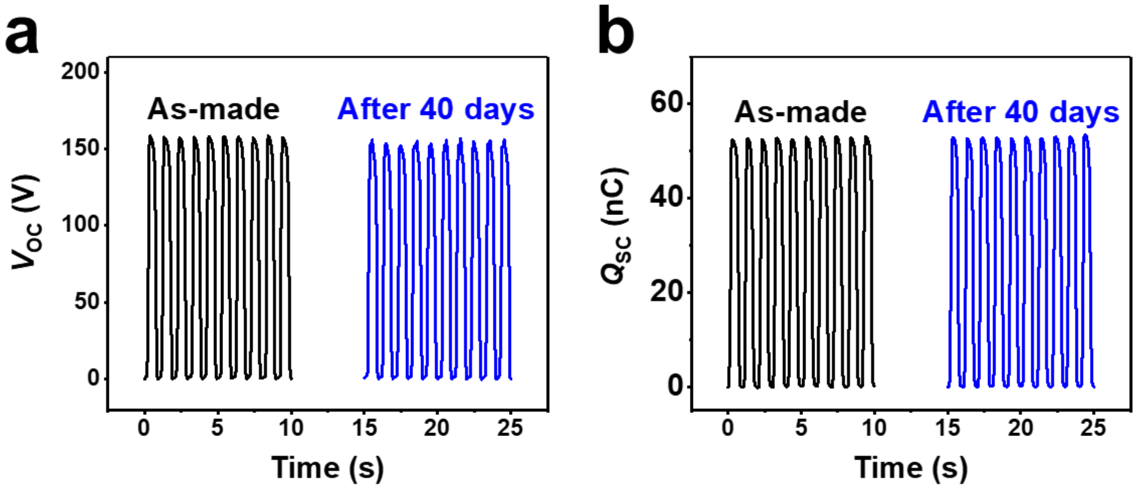

Fig. S37 Comparison of V_{OC} and Q_{SC} of CTA-TENG before and after storage in ambient environment for at least 40 days

S4 Supplementary Tables

Table S1 Effect of molar ratio on the properties of CECT

Sample	Feed ratio[a)]	DS_{COONa}[b)]	Water-solubility
CECT-5	5:1	0.51	soluble
CECT-7	7:1	0.58	soluble
CECT-9	9:1	0.69	soluble
CECT-11	11:1	0.75	soluble
CECT-13	13:1	0.86	soluble

a) Molar ratio of acrylamide to glucose units; b) Calculated from ^1H NMR

Table S2 Preparation and physical properties of CTA hydrogels

Sample	DS_{COONa} [a)]	c_{CECT} (wt%)	c_{AAm} (wt%)	Water content (wt%)	Tension				Compression			
					σ_b (kPa)	ε_b (%)	E (kPa)	W_f (kJ m^{-3})	σ_b (MPa)	ε_b (%)	E (kPa)	W_f (kJ m^{-3})
$C_0T_0A_{20}$	0	0	20	74.8	57.65	735.43	30.93	227.72	0.68	90	16.75	44.98
$C_5T_4A_{20}$	0.51	4	20	67.4	213.93	1157.73	60.69	1031.74	2.93	90	43.59	156.54
$C_7T_4A_{20}$	0.58	4	20	68.7	213.78	1291.52	47.59	873.73	2.87	90	30.19	175.62
$C_9T_4A_{20}$	0.69	4	20	70.3	186.41	1252.12	45.15	739.70	2.76	90	28.15	157.85
$C_{11}T_4A_{20}$	0.75	4	20	69.7	192.67	1585.77	43.26	1299.71	1.31	90	19.35	77.43
$C_{13}T_4A_{20}$	0.86	4	20	69.6	191.15	1307.11	53.75	1030.17	2.89	90	33.09	173.32
$C_{11}T_2A_{20}$	0.75	2	20	73.58	121.26	1109.54	39.16	498.93	0.97	90	13.45	59.48
$C_{11}T_6A_{20}$	0.75	6	20	69.60	226.84	1090.83	54.67	838.47	2.38	90	49.82	139.54
$C_{11}T_8A_{20}$	0.75	8	20	66.50	140.74	835.57	66.62	505.57	2.96	90	52.92	153.45
$C_{11}T_4A_{15}$	0.75	4	15	76.60	195.32	1341.41	24.98	924.08	0.38	90	9.76	24.65
$C_{11}T_4A_{25}$	0.75	4	25	66.10	184.71	852.57	90.28	671.33	3.32	90	57.77	248.94

a) Calculated from ^{1}H NMR

$\boldsymbol{\sigma_b}$ is the stress. $\boldsymbol{\varepsilon_b}$ is the strain. **E** is the Young's modulus. $\mathbf{W_f}$ is the fracture energy

Table S3 Comparison of conductivity of CTA hydrogel and the reported conductive composites

Materials	Conductive medium	Conductivity (S m^{-1})	Refs.
A-MXene/D-PDMS	A-MXene	5.27×10^{-2}	[S23]
TPU/CNCTT	CNT	1×10^{-2}	[S24]
HSAH/PHEAA	DESs	0.19	[S25]
ELO/PANI	PANI	8.64×10^{-4}	[S26]
AHS	SBMA/HEMA	0.39	[S12]
PEDOT:PSS-PAAm	PEDOT:PSS-PAAm	1.08×10^{-2}	[S27]
NAGA-*co*-AAm/LiCl	Li^+, Cl^-	0.69	[S28]
XSBR/SSCNT	CNT	7.08×10^{-2}	[S29]
ICE	[Emim][OAc]	0.77×10^{-2}	[S30]
PTCM-Gly5	MXene	0.19	[S31]
Ionogel-HPC	[Bmin]Cl	10.2×10^{-3}	[S32]
MMCOH	CNT	1×10^{-3}	[S33]
MASTA-PANI$_5$	PANI	7.8×10^{-4}	[S34]
Ionogel-4050	[EMIM][TFSI]	0.29	[S35]
CPH	$BzMe_3NOH$	0.46	[S36]
**CTAs**	**Na^+, K^+, OH^-**	**0.62**	**This work**

Table S4 A table lists the letters corresponding to International Morse code

Code	Letter	Code	Letter	Code	Letter	Code	Letter
• —	A	• • • •	H	— — —	O	• • • —	V
— • • •	B	• •	I	• — — •	P	• — —	W
— • — •	C	• — — —	J	— — • —	Q	— • • —	X
— • •	D	— • —	K	• — •	R	— • — —	Y
•	E	• — • •	L	• • •	S	— — • •	Z
• • — •	F	— —	M	—	T		
— — •	G	— •	N	• • —	U		

Table S5 Comparison of the electrical output of the TENG device with previous literature

Literature	Friction materials	Fre (Hz)	*P*	Refs.
Yao et al.	CNFs-FEP	10	140 mW/m^2	[S37]
Gao et al.	PEDOT:PSS-PLA	5	211 mW/m^2	[S38]
Yeh et al.	Ecoflex-Liquid metal	/	19.04 mW/m^2	[S39]
Kim et al.	PTFE-Al/Tin	/	147 mW/m^2	[S40]
Zheng et al.	PLGA-PCL	1	32.6 mW/m^2	[S41]
Wang et al.	Chitosan-Ecoflex	0.5	17.5 μW/m^2	[S42]
Pang et al.	Alginate-Al	1	9.5 μW	[S43]
Pan et al.	PLA-Gelatin	5	5 W/m^2	[S44]
Liang et al.	SA-PVA	1	3.8 mW/m^2	[S45]
S. Parandeh et al.	Paper-PCL/GO	3	72.5 mW/m^2	[S46]
Sriphan et al.	Ti_2NbO_7 NSs-BC	/	28 μW	[S47]
Lu et al.	Sugar-Nickel	4	4.21 W/m^2	[S48]
Wu et al.	PBS-SR	5	1.25 W/m^2	[S50]
Chi et al.	Rice paper-PVC	5	376 mW/m^2	[S51]
Zhang et al.	Chitin-VHB	3	1.25 W/m^2	[S52]
Yang et al.	Weighing paper-PTFE	4	13 mW	[S53]
/	***Latex-VHB***	***1***	***1.17 W/m^2***	***This work***

Supplementary References

[S1] S.D. Li, N. Chen, X.P. Li, Y. Li, Z. Xie et al., Bioinspired double-dynamic-bond crosslinked bioadhesive enables post-wound closure care. Adv. Funct. Mater. **30**(17), 2000130 (2020). https://doi.org/10.1002/adfm.202000130

[S2] D.L. Gan, W.S. Xing, L.L. Jiang, J. Fang, C.C. Zhao et al., Plant-inspired adhesive and

tough hydrogel based on ag-lignin nanoparticles-triggered dynamic redox catechol chemistry. Nat. Commun. **10**, 1487 (2019). https://doi.org/10.1038/s41467-019-09351-2

[S3] X. Su, Y. Luo, Z.L. Tian, Z.Y. Yuan, Y.M. Han et al., Ctenophore-inspired hydrogels for efficient and repeatable underwater specific adhesion to biotic surfaces. Mater. Horiz. **7**(10), 2651-2661 (2020). https://doi.org/10.1039/d0mh01344g

[S4] L. Han, L.W. Yan, M.H. Wang, K.F. Wang, L.M. Fang et al., Transparent, adhesive, and conductive hydrogel for soft bioelectronics based on light-transmitting polydopamine-doped polypyrrole nanofibrils. Chem. Mater. **30**(16), 5561-5572 (2018). https://doi.org/10.1021/acs.chemmater.8b01446

[S5] Y. Qian, Y.J. Zhou, M.J. Lu, X.S. Guo, D.J. Yang et al., Direct construction of catechol lignin for engineering long-acting conductive, adhesive, and UV-blocking hydrogel bioelectronics. Small Methods **5**(5), 2001311 (2021). https://doi.org/10.1002/smtd.202001311

[S6] X. Jing, H.Y. Mi, Y.J. Lin, E. Enriquez, X.F. Peng et al., Highly stretchable and biocompatible strain sensors based on mussel-inspired super-adhesive self-healing hydrogels for human motion monitoring. ACS Appl. Mater. Interfaces **10**(24), 20897-20909 (2018). https://doi.org/10.1021/acsami.8b06475

[S7] L. Han, K.Z. Liu, M.H. Wang, K.F. Wang, L.M. Fang et al., Mussel-inspired adhesive and conductive hydrogel with long-lasting moisture and extreme temperature tolerance. Adv. Funct. Mater. **28**(3), 1704195 (2018). https://doi.org/10.1002/adfm.201704195

[S8] Q.J. Fu, S.W. Hao, L. Meng, F. Xu, J. Yang, Engineering self-adhesive polyzwitterionic hydrogel electrolytes for flexible zinc-ion hybrid capacitors with superior low-temperature adaptability. ACS Nano **15**(11), 18469-18482 (2021). https://doi.org/10.1021/acsnano.1c08193

[S9] J.P. Liu, P. Zhang, H.Q. Wei, Z. Lu, Y. Yu, Printable tough adhesive for instant fatigue-resistant bonding of diverse surfaces. Adv. Funct. Mater. **32**(4), 2107732 (2022). https://doi.org/10.1002/adfm.202107732

[S10] G.X. Pan, F.H. Li, S.H. He, W.D. Li, Q.M. Wu et al., Mussel- and barnacle cement proteins-inspired dual-bionic bioadhesive with repeatable wet-tissue adhesion, multimodal self-healing, and antibacterial capability for nonpressing hemostasis and promoted wound healing. Adv. Funct. Mater. **32**(25), 2200908 (2022). https://doi.org/10.1002/adfm.202200908

[S11] Z.F. Yang, R.K. Huang, B.N. Zheng, W.T. Guo, C.K. Li et al., Highly stretchable, adhesive, biocompatible, and antibacterial hydrogel dressings for wound healing. Adv. Sci. **8**(8), 2003627 (2021). https://doi.org/10.1002/advs.202003627

[S12] H. Wei, Z. Wang, H. Zhang, Y. Huang, Z. Wang et al., Ultrastretchable, highly transparent, self-adhesive, and 3d-printable ionic hydrogels for multimode tactical sensing. Chem. Mater. **33**(17), 6731-6742 (2021). https://doi.org/10.1021/acs.chemmater.1c01246

[S13] K. Meng, S. Zhao, Y. Zhou, Y. Wu, S. Zhang et al., A wireless textile-based sensor system for self-powered personalized health care. Matter **2**(4), 896-907 (2020). https://doi.org/10.1016/j.matt.2019.12.025

[S14] Y. Guo, M. Zhong, Z. Fang, P. Wan, G. Yu, A wearable transient pressure sensor made with MXene nanosheets for sensitive broad-range human–machine interfacing. Nano Lett. **19**(2), 1143-1150 (2019). https://doi.org/10.1021/acs.nanolett.8b04514

[S15] F. Guan, Y. Xie, H. Wu, Y. Meng, Y. Shi et al., Silver nanowire–bacterial cellulose

composite fiber-based sensor for highly sensitive detection of pressure and proximity. ACS Nano **14**(11), 15428-15439 (2020). https://doi.org/10.1021/acsnano.0c06063

[S16] M. Jian, K. Xia, Q. Wang, Z. Yin, H. Wang et al., Flexible and highly sensitive pressure sensors based on bionic hierarchical structures. Adv. Funct. Mater. **27**(9), 1606066 (2017). https://doi.org/10.1002/adfm.201606066

[S17] G. Ge, Y. Zhang, J. Shao, W. Wang, W. Si et al., Stretchable, transparent, and self-patterned hydrogel-based pressure sensor for human motions detection. Adv. Funct. Mater. **28**(32), 1802576 (2018). https://doi.org/10.1002/adfm.201802576

[S18] Z. Shen, X. Zhu, C. Majidi, G. Gu, Cutaneous ionogel mechanoreceptors for soft machines, physiological sensing, and amputee prostheses. Adv. Mater. **33**(38), 2102069 (2021). https://doi.org/10.1002/adma.202102069

[S19] J.J. Lee, S. Gandla, B. Lim, S. Kang, S. Kim et al., Alcohol-based highly conductive polymer for conformal nanocoatings on hydrophobic surfaces toward a highly sensitive and stable pressure sensor. NPG Asia Mater. **12**(1), 65 (2020). https://doi.org/10.1038/s41427-020-00238-z

[S20] X. Pu, M. Liu, X. Chen, J. Sun, C. Du et al., Ultrastretchable, transparent triboelectric nanogenerator as electronic skin for biomechanical energy harvesting and tactile sensing. Sci. Adv. **3**(5), 1700015 (2017). https://doi.org/10.1126/sciadv.1700015

[S21] Y. Wang, H. Wu, L. Xu, H. Zhang, Y. Yang et al., Hierarchically patterned self-powered sensors for multifunctional tactile sensing. Sci. Adv. **6**(34), 9083 (2020). https://doi.org/10.1126/sciadv.abb9083

[S22] S. Lee, S. Franklin, F.A. Hassani, T. Yokota, O.G. Nayeem et al., Nanomesh pressure sensor for monitoring finger manipulation without sensory interference. Science **370**(6519), 966-970 (2020). https://doi.org/10.1126/science.abc9735

[S23] K. Zhang, J. Sun, J. Song, C. Gao, Z. Wang et al., Self-healing Ti_3C_2 MXene/PDMS supramolecular elastomers based on small biomolecules modification for wearable sensors. ACS Appl. Mater. Interfaces **12**(40), 45306-45314 (2020). https://doi.org/10.1021/acsami.0c13653

[S24] Z. Sang, K. Ke, I. Manas-Zloczower, Effect of carbon nanotube morphology on properties in thermoplastic elastomer composites for strain sensors. Compos. Part A Appl. Sci. Manuf. **121**, 207-212 (2019). https://doi.org/10.1016/j.compositesa.2019.03.007

[S25] Y. Liang, K. Wang, J. Li, H. Wang, X.Q. Xie et al., Low-molecular-weight supramolecular-polymer double-network eutectogels for self-adhesive and bidirectional sensors. Adv. Funct. Mater. **31**(45), 2104963 (2021). https://doi.org/10.1002/adfm.202104963

[S26] V. Khandelwal, S.K. Sahoo, A. Kumar, G. Manik, Electrically conductive green composites based on epoxidized linseed oil and polyaniline: an insight into electrical, thermal and mechanical properties. Compos. Part B Eng. **136**, 149-157 (2018). https://doi.org/10.1016/j.compositesb.2017.10.030

[S27] Y.Y. Lee, H.Y. Kang, S.H. Gwon, G.M. Choi, S.M. Lim et al., A strain-insensitive stretchable electronic conductor: PEDOT:PSS/acrylamide organogels. Adv. Mater. **28**(8), 1636-1643 (2016). https://doi.org/10.1002/adma.201504606

[S28] L. Shuai, Z.H. Guo, P. Zhang, J. Wan, X. Pu et al., Stretchable, self-healing, conductive hydrogel fibers for strain sensing and triboelectric energy-harvesting smart textiles. Nano Energy **78**, 105389 (2020). https://doi.org/10.1016/j.nanoen.2020.105389

[S29] M. Lin, Z. Zheng, L. Yang, M. Luo, L. Fu et al., A high-performance, sensitive, wearable multifunctional sensor based on rubber/CNT for human motion and skin temperature detection. Adv. Mater. **34**(1), 2107309 (2022). https://doi.org/10.1002/adma.202107309

[S30] C. Dang, F. Peng, H. Liu, X. Feng, Y. Liu et al., Facile solvent-free synthesis of multifunctional and recyclable ionic conductive elastomers from small biomass molecules for green wearable electronics. J. Mater. Chem. A **9**(22), 13115-13124 (2021). https://doi.org/10.1039/d1ta01659h

[S31] Y. Wei, L. Xiang, H. Ou, F. Li, Y. Zhang et al., MXene-based conductive organohydrogels with long-term environmental stability and multifunctionality. Adv. Funct. Mater. **30**(48), 2005135 (2020). https://doi.org/10.1002/adfm.202005135

[S32] C. Dang, C. Shao, H. Liu, Y. Chen, H. Qi, Cellulose melt processing assisted by small biomass molecule to fabricate recyclable ionogels for versatile stretchable triboelectric nanogenerators. Nano Energy **90**, 106619 (2021). https://doi.org/10.1016/j.nanoen.2021.106619

[S33] H. Sun, Y. Zhao, S. Jiao, C. Wang, Y. Jia et al., Environment tolerant conductive nanocomposite organohydrogels as flexible strain sensors and power sources for sustainable electronics. Adv. Funct. Mater. **31**(24), 2101696 (2021). https://doi.org/10.1002/adfm.202101696

[S34] K.X. Hou, S.P. Zhao, D.P. Wang, P.C. Zhao, C.H. Li et al., A puncture-resistant and self-healing conductive gel for multifunctional electronic skin. Adv. Funct. Mater. **31**(49), 2107006 (2021). https://doi.org/10.1002/adfm.202107006

[S35] L. Xu, Z. Huang, Z. Deng, Z. Du, T.L. Sun et al., A transparent, highly stretchable, solvent-resistant, recyclable multifunctional ionogel with underwater self-healing and adhesion for reliable strain sensors. Adv. Mater. **33**(51), 2105306 (2021). https://doi.org/10.1002/adma.202105306

[S36] Y. Wang, L. Zhang, A. Lu, Highly stretchable, transparent cellulose/PVA composite hydrogel for multiple sensing and triboelectric nanogenerators. J. Mater. Chem. A **8**(28), 13935-13941 (2020). https://doi.org/10.1039/d0ta02010a

[S37] C. Yao, A. Hernandez, Y. Yu, Z. Cai, X. Wang, Triboelectric nanogenerators and power-boards from cellulose nanofibrils and recycled materials. Nano Energy **30**, 103-108 (2016). https://doi.org/10.1016/j.nanoen.2016.09.036

[S38] X. Gao, L. Huang, B. Wang, D. Xu, J. Zhong et al., Natural materials assembled, biodegradable, and transparent paper-based electret nanogenerator. ACS Appl. Mater. Interfaces **8**(51), 35587-35592 (2016). https://doi.org/10.1021/acsami.6b12913

[S39] C. Yeh, F.C. Kao, P.H. Wei, A. Pal, K. Kaswan et al., Bioinspired shark skin-based liquid metal triboelectric nanogenerator for self-powered gait analysis and long-term rehabilitation monitoring. Nano Energy **104**, 107852 (2022). https://doi.org/10.1016/j.nanoen.2022.107852

[S40] Y.W. Kim, S. Akin, H. Yun, S. Xu, W. Wu et al., Enhanced performance of triboelectric nanogenerators and sensors via cold spray particle deposition. ACS Appl. Mater. Interfaces **14**(41), 46410-46420 (2022). https://doi.org/10.1021/acsami.2c09367

[S41] Q. Zheng, Y. Zou, Y. Zhang, Z. Liu, B. Shi et al., Biodegradable triboelectric nanogenerator as a life-time designed implantable power source. Sci. Adv. **2**(3), 1501478 (2016). https://doi.org/10.1126/sciadv.1501478

[S42] R. Wang, S. Gao, Z. Yang, Y. Li, W. Chen et al., Engineered and laser-processed

chitosan biopolymers for sustainable and biodegradable triboelectric power generation. Adv. Mater. **30**(11), 1706267 (2018). https://doi.org/10.1002/adma.201706267

[S43] Y. Pang, F. Xi, J. Luo, G. Liu, T. Guo et al., An alginate film-based degradable triboelectric nanogenerator. RSC Adv. **8**(12), 6719-6726 (2018). https://doi.org/10.1039/c7ra13294h

[S44] R. Pan, W. Xuan, J. Chen, S. Dong, H. Jin et al., Fully biodegradable triboelectric nanogenerators based on electrospun polylactic acid and nanostructured gelatin films. Nano Energy **45**, 193-202 (2018). https://doi.org/10.1016/j.nanoen.2017.12.048

[S45] Q. Liang, Q. Zhang, X. Yan, X. Liao, L. Han et al., Recyclable and green triboelectric nanogenerator. Adv. Mater. **29**(5), 1604961 (2017). https://doi.org/10.1002/adma.201604961

[S46] S. Parandeh, M. Kharaziha, F. Karimzadeh, An eco-friendly triboelectric hybrid nanogenerators based on graphene oxide incorporated polycaprolactone fibers and cellulose paper. Nano Energy **59**, 412-421 (2019). https://doi.org/10.1016/j.nanoen.2019.02.058

[S47] S. Sriphan, U. Pharino, T. Charoonsuk, P. Pulphol, P. Pakawanit et al., Tailoring charge affinity, dielectric property, and band gap of bacterial cellulose paper by multifunctional Ti_2NbO_7 nanosheets for improving triboelectric nanogenerator performance. Nano Res., (2022). https://doi.org/10.1007/s12274-022-4957-3

[S48] H. Lu, W. Zhao, Z.L. Wang, X. Cao, Sugar-based triboelectric nanogenerators for effectively harvesting vibration energy and sugar quality assessment. Nano Energy **88**, 106196 (2021). https://doi.org/10.1016/j.nanoen.2021.106196

[S49] G. Khandelwal, T. Minocha, S.K. Yadav, A. Chandrasekhar, N.P.M.J. Raj et al., All edible materials derived biocompatible and biodegradable triboelectric nanogenerator. Nano Energy **65**, 104016 (2019). https://doi.org/10.1016/j.nanoen.2019.104016

[S50] Y. Wu, Y. Luo, J. Qu, W.A. Daoud, T. Qi, Nanogap and environmentally stable triboelectric nanogenerators based on surface self-modified sustainable films. ACS Appl. Mater. Interfaces **12**(49), 55444-55452 (2020). https://doi.org/10.1021/acsami.0c16671

[S51] Y. Chi, K. Xia, Z. Zhu, J. Fu, H. Zhang et al., Rice paper-based biodegradable triboelectric nanogenerator. Microelectron. Eng. **216**, 111059 (2019). https://doi.org/10.1016/j.mee.2019.111059

[S52] J. Zhang, Y. Hu, X. Lin, X. Qian, L. Zhang et al., High-performance triboelectric nanogenerator based on chitin for mechanical-energy harvesting and self-powered sensing. Carbohydrate Polym. **291**, 119586 (2022). https://doi.org/10.1016/j.carbpol.2022.119586

[S53] Y. Yang, D. Zhang, D. Wang, Z. Xu, J. Zhang, A high-stability weighing paper/polytetrafluoroethylene-based triboelectric nanogenerator for self-powered In_2O_3 nanocubes/SnS_2 nanoflower NO_2 gas sensors. J. Mater. Chem. A **9**(25), 14495-14506 (2021). https://doi.org/10.1039/d1ta03739k
